# Designing Red-Shifted Molecular Emitters Based on the Annulated Locked GFP Chromophore Derivatives

**DOI:** 10.3390/ijms222413645

**Published:** 2021-12-20

**Authors:** Gregory D. Sinenko, Dilara A. Farkhutdinova, Ivan N. Myasnyanko, Nadezhda S. Baleeva, Mikhail S. Baranov, Anastasia V. Bochenkova

**Affiliations:** 1 Department of Chemistry, Lomonosov Moscow State University, Leninskie Gory 1/3, 119991 Moscow, Russia; grigorisin@gmail.com (G.D.S.); farkhutdinovada@gmail.com (D.A.F.); 2 Institute of Bioorganic Chemistry, Russian Academy of Sciences, Miklukho-Maklaya 16/10, 117997 Moscow, Russia; conzbutcher@gmail.com (I.N.M.); nsbaleeva@gmail.com (N.S.B.); baranovmikes@gmail.com (M.S.B.); 3 Institute of Translational Medicine, Pirogov Russian National Research Medical University, Ostrovitianov 1, 117997 Moscow, Russia

**Keywords:** GFP chromophore, polycyclic aromatic hydrocarbons, red-shifted molecular emitters, multi-state multi-reference perturbation theory

## Abstract

Bioimaging techniques require development of a wide variety of fluorescent probes that absorb and emit red light. One way to shift absorption and emission of a chromophore to longer wavelengths is to modify its chemical structure by adding polycyclic aromatic hydrocarbon (PAH) fragments, thus increasing the conjugation length of a molecule while maintaining its rigidity. Here, we consider four novel classes of conformationally locked Green Fluorescent Protein (GFP) chromophore derivatives obtained by extending their aromatic systems in different directions. Using high-level ab initio quantum chemistry calculations, we show that the alteration of their electronic structure upon annulation may unexpectedly result in a drastic change of their fluorescent properties. A flip of optically bright and dark electronic states is most prominent in the symmetric fluorene-based derivative. The presence of a completely dark lowest-lying excited state is supported by the experimentally measured extremely low fluorescence quantum yield of the newly synthesized compound. Importantly, one of the asymmetric modes of annulation provides a very promising strategy for developing red-shifted molecular emitters with an absorption wavelength of ∼600 nm, having no significant impact on the character of the bright S-S_1_ transition.

## 1. Introduction

Today, the importance of fluorescent labeling in visualization of biological processes is well established [1,2]. Most valuable properties of fluorescent probes include high fluorescence quantum yields (FQYs) and red-shifted absorption and emission wavelengths. While the former is obviously important to increase signal-to-noise ratio, the latter is proved to be useful for deep tissue imaging, minimizing autofluorescence and reducing damaging effects of light [3,4,5]. Absorption and emission in near-infrared transparency window of biological tissues are, therefore, highly advantageous for bioimaging.

Synthetic organic small π-conjugated fluorophores have received special interest as potential fluorescent labels owing to their adjustable selectivity/sensitivity, as well as relatively inexpensive synthesis [2,6]. Among these, derivatives of the Green Fluorescent Protein (GFP), which are represented by a diverse library of the benzylidene imidazolone (BDI) core [7], are intensively being studied [8,9].

It is well known that methine dyes, such as BDI derivatives, exhibit significant loss of FQY caused by facile rotation in the central methine bridge, which results in nonradiative decay through internal conversion [10,11,12,13]. However, intramolecular rotation upon photoexcitation can be arrested. One way to fix the arylidene fragment is to introduce the difluoroboryl bridge into the chemical structure of BDI chromophores. Indeed, fluorescence is recovered in various locked GFP derivatives [14,15,16,17,18].

While high FQYs of chromophores are necessary for bioimaging applications, the search for molecular emitters that absorb and emit red light is another important problem. The common approach is based on the extension of π-conjugation, and it has successfully been applied to many classes of chromophores by introducing π-conjugated substituents into the core of a molecule (see, for example, References [19,20,21]). The alternative way is to expand an aromatic system by annulation of its core [22,23,24,25]. Recently, this approach has been tested on GFP derivatives [15]. It is advantageous not only for red-shifting absorption and emission maxima, but also for increasing Stokes shifts of chromophores, which is important for multi-color labeling applications [26,27]. However, the mode of annulation is found to play an important role in the photophysical properties of previously synthesized dyes [28,29,30]. For some compounds, FQYs demonstrate a tendency to considerably decrease upon annulation [31,32]. Therefore, care should be taken when increasing the size of a conjugated system: while the position of the lowest-lying absorption is expected to shift toward lower energies, the corresponding oscillator strengths may become negligible. In this regard, computer-aided design might be very helpful in predicting the best direction for annulation.

**Figure 1:**
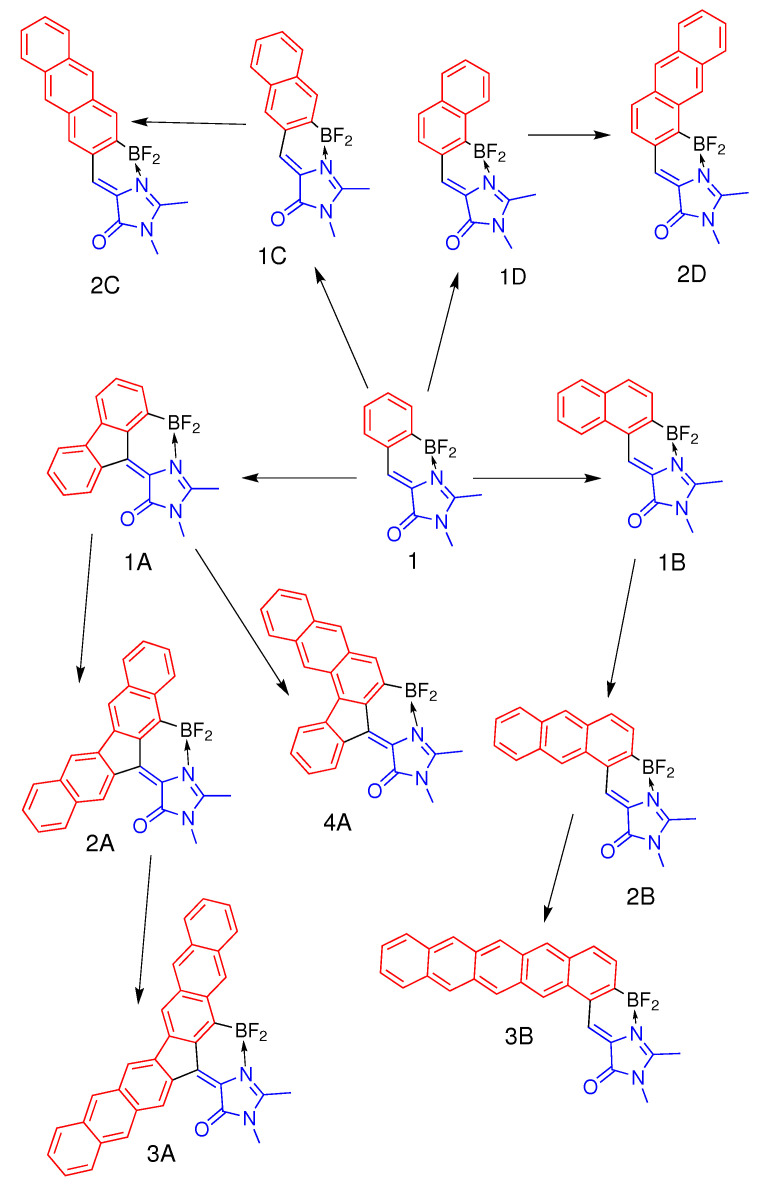
Four structural classes of the annulated locked GFP chromophores: **A**, **B**, **C**, and **D**. Fluorene-based compound **1A** has been synthesized and characterized in this work, while compounds **1** and **1B** have been synthesized and characterized previously [14,15].The GFP chromophore’s core (subsystem I), which includes imidazolinone and the methine bridge double bond, is depicted in blue, and annulated PAHs (subsystem II) are highlighted in red.

Here, by using high-level ab initio quantum chemistry calculations, we explore various ways of polycyclic modifications of the previously synthesized BF_2_-locked GFP-derivative [14] (compound **1** in Figure 1) to gain the red-shifted absorption and emission. We consider four novel classes by combining the molecular core of compound **1** with polycyclic aromatic hydrocarbons (PAHs) shown in Figure 1. We show that different modes of annulation provide different patterns of the interplay between electronic structures of the GFP chromophore’s core and PAHs, designated as subsystems I and II, respectively, and shown in Figure 1. Remarkably, a flip of optically bright and dark states may occur upon certain modes of annulation. This feature is unexpectedly most prominent in the symmetric small fluorene-based GFP derivative (compound **1A** in Figure 1). To support our findings, we synthesize compound **1A** and demonstrate that it is indeed non-fluorescent. Finally, we reveal that one of the asymmetric modes of annulation is promising for theory-driven synthesis of red-shifted molecular emitters based on the GFP chromophore modified by PAHs.

## 2. Materials and Methods

### 2.1. Experimental Details

Commercially available reagents were used without additional purification. Merck Kieselgel 60 (Darmstadt, Germany) was used for column chromatography. Thin-layer chromatography (TLC) was performed on silica gel 60 F_254_ glass-backed plates (MERCK). Visualization was performed using UV light (254 or 312 nm) and staining with KMnO_4_.

NMR spectra were recorded on a 700 MHz Bruker Avance III NMR (Rheinstetten, Germany) at 303 K. Chemical shifts are reported relative to residue peaks of DMSO-d6 (2.51 ppm for ${}^{1}$H and 39.5 ppm for ${}^{13}$C). Melting points were measured on an SMP 30 apparatus without correction. High-resolution mass spectra (HRMS) were recorded on a Bruker micrOTOF II instrument and AB Sciex TripleTOF${}^{®}$ 5600${}^{+}$ System using electrospray ionization (ESI). The measurements were done in a positive ion mode (interface capillary voltage—5500 V on TripleTOF${}^{®}$ and 4500 V on micrOTOF II) or in a negative ion mode (4500 V on TripleTOF${}^{®}$; 3200 V on micrOTOF II); mass range from *m*/*z* 50 to *m*/*z* 3000; external or internal calibration was done with ESI Tuning Mix, Agilent (Santa-Clara, CA, USA). A syringe injection was used for solutions in acetonitrile, methanol, or water (flow rate 20 μ/min on TripleTOF${}^{®}$; 3 mL/min on micrOTOF II). Nitrogen was applied as a dry gas; interface temperature was set at 180 °C.

UV-VIS spectra were recorded with a Varian Cary 100 spectrophotometer (Melbourne, Australia). Fluorescence emission spectra were recorded with Agilent Cary Eclipse fluorescence spectrophotometer (Melbourne, Australia).

**Scheme 1:**
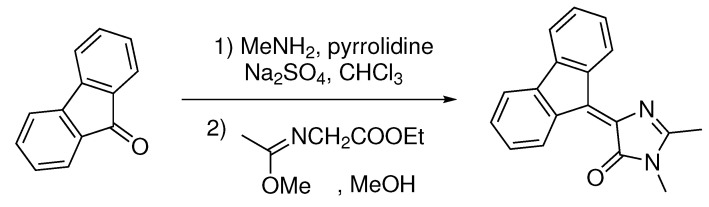
Synthesis of 4-(9H-fluoren-9-ylidene)-1,2-dimethyl-1H-imidazol-5(4H)-one.

*Synthesis of 4-(9H-fluoren-9-ylidene)-1,2-dimethyl-1H-imidazol-5(4H)-one*. 9H-fluoren-9-one (1.8 g, 10 mmol) was dissolved in CHCl_3_ (75 mL) and mixed with methylamine solution (40% aqueous, 3.0 mL), pyrrolidine (30 mg, 0.5 mmol), and anhydrous Na_2_SO_4_ (20 g). The mixture was stirred for 28 days at room temperature, filtered and dried over the additional Na_2_SO_4_. The solvent was evaporated, and ethyl-((methoxy)amino)acetate (1.9 g, 12 mmol) was added to the residue. The mixture was stirred for 7 days at room temperature, solvents were evaporated, and the product was purified by column chromatography (EtOAc:Hexane 1:3) (Scheme 1).

Yellow solid (1.51 g, 55%); M.p. 173–175 °C; ${}^{1}$H NMR (700 MHz, DMSO-d6) δ ppm 9.40 (d, J = 7.8 Hz, 1H), 8.98 (d, J = 7.8 Hz, 1H), 7.84 (d, J = 7.4 Hz, 1H), 7.81 (d, J = 7.4 Hz, 1H), 7.45 (t, J = 7.4 Hz, 1H), 7.41 (t, J = 7.4 Hz, 1H), 7.36–7.31 (m, 2H), 3.18 (s, 3H), 2.45 (s, 3H); ${}^{13}$C NMR (75 MHz, DMSO-d6) δ ppm 168.8, 164.0, 141.2, 141.1, 139.0, 138.0, 135.7, 135.4, 130.7, 130.5, 130.2, 128.2, 127.9, 127.6, 120.0, 119.8, 26.5, 15.6; HRMS (ESI) *m*/*z*: 275.1181 found (calcd for C_18_H_15_N_2_O^+^, [M+H]${}^{+}$ 275.1184).

**Scheme 2:**
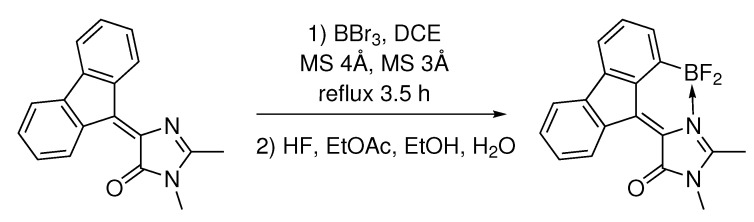
Synthesis of (Z)-4-(1-(difluoroboryl)-9H-fluoren-9-ylidene)-1,2-dimethyl-1H-imidazol-5(4H)-one (compound **1A**).

*Synthesis of (Z)-4-(1-(difluoroboryl)-9H-fluoren-9-ylidene)-1,2-dimethyl-1H-imidazol-5(4H)-one* (compound **1A**). 4-(9H-fluoren-9-ylidene)-1,2-dimethyl-1H-imidazol-5(4H)-one (550 mg, 2 mmol) was dissolved in freshly distilled DCE (200 mL). A flask was loaded with commercially available molecular sieves (30 g 3 Å and 30 g 4 Å) and purged with argon. Solution of BBr_3_ (1 M in DCM, 20 mL, 20 mmol) was added, and the reaction mixture was refluxed for 6 h and left to stir at room temperature overnight. Molecular sieves were filtered, thoroughly washed with ethanol (100 mL) and ethyl acetate (100 mL). To the filtrate HF was added (20% in water, 20 mL), and the mixture was stirred for 30 min, then diluted with water (100 mL), and quenched with solid sodium bicarbonate until pH = 7. The reaction mixture was transferred to a separatory funnel and diluted with ethyl acetate (200 mL). The organic phase was separated and washed with brine (3 × 100 mL) and was dried over Na_2_SO_4_. The solvents were evaporated and the crude product was purified by flash column chromatography (DCM:MeOH 100:1) (Scheme 2).

Yellow solid (550 mg, 85%); M.p. over 220 °C with decomposition; ${}^{1}$H NMR (700 MHz, DMSO-d6) δ ppm 8.82 (d, J = 7.6 Hz, 1H), 7.82 (d, J = 7.4 Hz, 1H), 7.67 (d, J = 6.9 Hz, 1H), 7.52 (t, J = 7.4 Hz, 1H), 7.44–7.36 (m, 3H), 3.32 (s, 3H), 2.84 (s, 3H); ${}^{13}$C NMR (75 MHz, DMSO-d6) δ ppm 167.9, 162.7, 143.4, 138.2, 137.5, 137.1, 133.3, 132.9, 132.0, 129.9, 128.5, 128.2, 123.5, 121.5, 119.9, 26.8, 13.3; HRMS (ESI) *m*/*z*: 303.1102 found (calcd for C_18_H_13_BFN_2_O^+^, [M-F]${}^{+}$ 303.1105).

### 2.2. Computational Details

For all quantum chemistry calculations, Firefly version 8.2.0 [33], partially based on the GAMESS (US) source code [34], was used. Ground-state equilibrium geometry parameters for all molecules were found at the MP2 level of theory (geometries are provided in the Supplementary Materials). The (aug)-cc-pVDZ basis set augmented with diffuse functions on oxygen and nitrogen atoms was used. Excited-state calculations were performed using the extended multi-configuration quasi-degenerate perturbation theory XMCQDPT2 [35].

Initially, all valence π-type orbitals were included in the active spaces of the analyzed molecules using the state-averaged SA(7)-MCSCF(1+2) method, which was restricted to single and double excitations within an active space with respect to a reference closed-shell configuration. We used the following active spaces: (14,13), i.e., 14 electrons distributed over 13 orbitals, for compound **1**, (20,19) for compound **1A**, (28,27) for compound **2A**, (36,35) for compound **3A**, (28,27) for compound **4A**, (18,17) for compound **1B**, (22,21) for compound **2B**, (30,29) for compound **3B**, (18,17) for compound **1C**, (22,21) for compound **2C**, (18,17) for compound **1D**, and (22,21) for compound **2D**. The active spaces were then reduced for the subsequent complete active space self-consistent field calculations at the SA(7)-CASSCF(14,14) level of theory. The reduction was done based on the analysis of occupation numbers of the natural SA(7)-MCSCF(1+2) orbitals. The orbitals with highest and lowest occupation numbers were excluded. Zero-order wave functions for the subsequent XMCQDPT2 perturbative treatment were obtained at the SA(7)-CASSCF(14,14) level of theory.

The XMCQDPT2 effective Hamiltonians were constructed within the model spaces spanned by 7 CASSCF zero-order wave functions. The zero-order wave functions were allowed to interact through effective Hamiltonian. Energies of perturbed states were obtained as eigenvalues of effective Hamiltonian, while projections of perturbed states onto zero-order states were defined by its eigenvectors. Energies of all semi-canonical orbitals used in the perturbative calculations were defined by eigenvalues of the corresponding blocks of the effective one-particle Fock operator, diagonalized in the basis of the computed CASSCF molecular orbitals. Minor changes in the zero-order CASSCF wave functions, obtained using some of the excluded SA(7)-MCSCF(1+2) natural orbitals, were found to have no significant impact on the results of the XMCQDPT2 calculations.

The eigenvectors of the effective Hamiltonian, obtained in the XMCQDPT2 calculations for compound **1A**, are presented in Table S1 in the Supplementary Materials. The S${}_{3}$ and S${}_{4}$ CASSCF reference states are found to have a significant contribution to the perturbed S${}_{2}$ state. Therefore, it is advisable to consider no less than 5 reference states in the XMCQDPT2 calculations to properly describe the S${}_{0}$–S${}_{2}$ transition for compound **1A**. The same problem of deficient description of the zero-order states obtained at the CASSCF level is observed for all molecules studied in this work. Therefore, the XMCQDPT2 method, which can improve the description of zero-order states, is required for calculations of vertical excitation energies in this case.

The constant contour value of 0.0015 was used for plotting differential electron densities Δρ obtained at the XMCQDPT2 level of theory. Chemcraft software [36] (Ivanovo, Russia) was used for visualizing Δρ and natural orbitals.

## 3. Results and Discussion

We explore four alternative strategies to extend the π-conjugated system of compound **1**. The first symmetric mode of annulation is based on the addition of fluorene-based PAHs to the central methine bridge, resulting in compounds **1A**, **2A**, and **3A** (Figure 1). Compound **4A** represents an asymmetric extension of fluorene-based compound **1A** by adding naphthalene only to one side of the molecule. The second symmetric mode of annulation is based on the extension of the benzene ring of subsystem II to naphthalene and anthracene, preserving effective axial symmetry of compound **1** and giving rise to compounds **1C** and **2C**. Finally, the two asymmetric modes include the addition of naphthalene, anthracene, and pentacene to the methine bridge in two different ways, which break effective symmetry, leading to compounds of classes **B** and **D** (Figure 1). The smallest-sized representatives of symmetric class **A** and asymmetric class **B** have been studied experimentally. Compound **1A** is synthesized and characterized in this work, whereas compound **1B**, as well as their predecessor, compound **1**, has been studied earlier [14,15].

The experimentally measured optical properties of compounds **1A** and **1B** are presented in Table 1 and compared to those of compound **1** (also see Figure S1 in the Supplementary Materials). In both cases, the first optically bright transition shifts to lower excitation energies upon annulation. However, the FQYs are found to be remarkably different in compounds **1A** and **1B**, despite their rigid structure, which prevents internal conversion through intramolecular twisting to take place. Indeed, compound **1B** is characterized by high FQYs in all solvents. At the same time, compound **1A** appears to be totally non-fluorescent with FQYs of less than 0.2% in all solvents. To disclose the origin of the unexpected photophysical properties of compound **1A**, we perform high level ab initio quantum chemistry calculations and then extend our computational study to all other compounds from classes **A**, **B**, **C**, and **D**.

We use the extended multi-configuration quasidegenerate perturbation theory (XMCQDPT2) [35] to calculate vertical excitation energies, oscillator strengths, and changes in average dipole moments upon excitation (Δμ) at the equilibrium geometry in the ground electronic state. The calculated photophysical properties of all compounds are presented in Table 2. We consider all low-energy transitions with appreciable oscillator strengths and all transitions that are below those. The calculated vertical excitation energies of the lowest-energy bright transitions are 3.21 eV (387 nm), 3.06 eV (405 nm), and 2.91 eV (426 nm) for compounds **1**, **1A**, and **1B**, respectively. These values are in good agreement with the experimental absorption maxima in dioxane, which are 3.40 eV (365 nm), 2.96 eV (419 nm), and 2.85 eV (435 nm) for compounds **1**, **1A**, and **1B**, respectively (see Table 1). The extremely low FQY of compound **1A**, which is found experimentally in all solvents, stems from the fact that its lowest-lying optically bright state appears to be the second S${}_{2}$ state, whereas the optical transitions involving the S${}_{1}$ state turn out to be completely dark. Indeed, the oscillator strength for the S${}_{0}$–S${}_{1}$ excitation is calculated to be on the order of 10${}^{-5}$. This is opposite to the order of states found in compounds **1** and **1B**, in which the S${}_{0}$–S${}_{1}$ transitions are the brightest.

A flip of dark and bright states can explain the unexpected drop in the FQY of compound **1A**. While excitation to the S${}_{2}$ state is allowed, internal conversion between electronically excited states often proceeds faster than radiative transitions; hence, fluorescence is expected to occur from the S${}_{1}$ state, which appears to be optically dark in compound **1A**. In many cases, fluorescence, if it is allowed and not outcompeted by nonradiative processes, occurs from the S${}_{1}$ state following intra- and intermolecular energy redistribution. This constitutes the so-called Kasha’s rule [37], and fluorescence does not usually depend on excitation wavelength. Compound **1A** obeys the Kasha’s rule, which prevents the radiative transition from the optically dark S${}_{1}$ state in this case. The questions then arise, why this flip of states happens and whether we can predict it when designing red-shifted molecular emitters.

Table 1 also demonstrates that emission shifts to longer wavelengths as solvent polarity increases, in particular for compound **1B**, where the solvent shift reaches a value of 0.24 eV in water as compared to dioxane. The computational results shown in Table 2 allow us to explain this trend by examining a charge-transfer (CT) character of the S${}_{0}$–S${}_{1}$ excitation. Indeed, the change in the average dipole moments upon the S${}_{0}$–S${}_{1}$ transition, $\Delta \mu (S_{1}$–$S_{0})$, is as high as 9.4 Debye, indicating a pronounced CT character of the first transition in compound **1B**. In compound **1**, a much lower value of the $\Delta \mu (S_{1}$–$S_{0})$ corresponds to its weaker $\lambda_{emission}$ dependence on solvent polarity, with the solvent shift being equal to 0.05 eV. Moreover, FQYs are also found to be solvent dependent and they generally have a tendency of becoming lower as solvent polarity increases. This also indicates a CT character of the first S${}_{0}$–S${}_{1}$ transition, which results in smaller energy gaps between the two states in polar solvents, thus enhancing radiationless transitions. However, knowledge of the detailed mechanisms of internal conversion and analysis of the influence of specific solvent effects on the electronic structure, such as H-bonding, are required to explain changes in FQYs of compound **1B** in particular solvents. At the same time, an extremely low emission intensity of compound **1A** makes it difficult to adequately estimate its $\lambda_{emission}$ and FQY dependence on solvent polarity.

In order to analyze and predict the potential effects of annulation, the XMCQDPT2 natural orbitals actively involved in the S${}_{0}$–S${}_{n}$ transitions are analyzed for all compounds. While, for n>2, the transitions demonstrate a complex structure with more than one pair of actively involved natural orbitals, the lowest-energy S${}_{0}$–S${}_{1}$ and S${}_{0}$–S${}_{2}$ transitions are both characterized by single predominant configurations, which refer to particular one-electron π-$\pi^{*}$ excitations, unless stated otherwise. This allows us to classify the lowest-energy transitions by two distinct types. These types are deduced by noting changes in electron density distribution and nodal structure of the corresponding molecular orbitals in the central methine bridge of the GFP chromophore’s core within subsystem I and analyzing the CT character of transitions in the coupled subsystems I and II (see Figure 1). To complement the analysis of electron density redistribution upon excitation using natural orbitals, we also consider differential one-particle electron densities between the states of interest based on calculated many-electron wavefunctions, Δρ.

**Figure 2:**
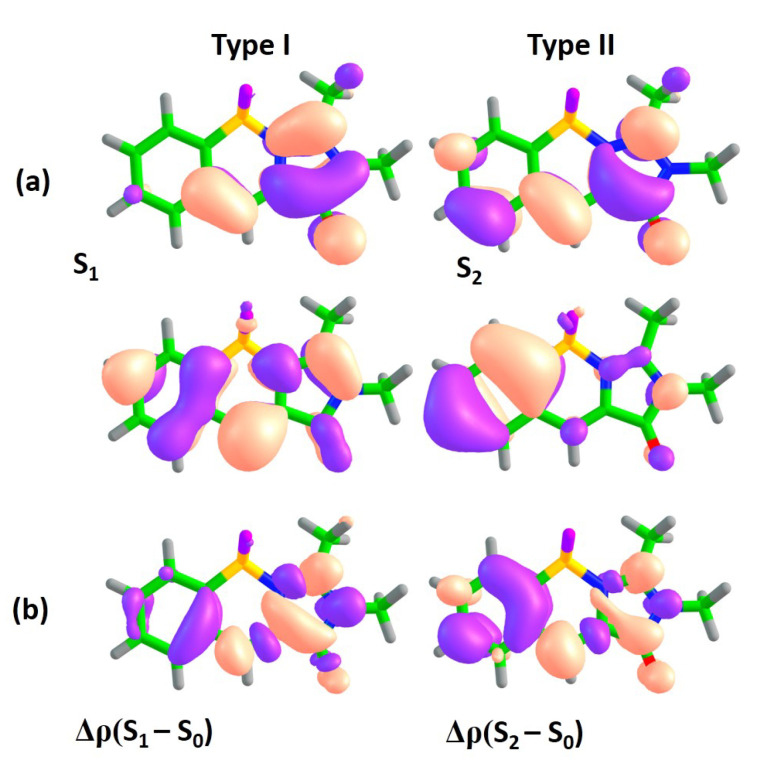
XMCQDPT2 natural orbitals actively involved in the S${}_{0}$–S${}_{1}$ and S${}_{0}$–S${}_{2}$ transitions (**a**) and the corresponding differential electron densities Δρ (**b**) for compound **1**. For each state, the excitation is directed from the lower orbital to the higher orbital. The color code stands for negative and positive Δρ values depicted in blue and red, respectively.

Figure 2a shows orbitals which are actively involved in the S${}_{0}$–S${}_{1}$ and S${}_{0}$–S${}_{2}$ transitions for compound **1**. The S${}_{0}$–S${}_{1}$ transition is accompanied by electron density redistribution in the central methine bridge of subsystem I. The C=C bridge bond acquires an anti-bonding character upon excitation, as it can be seen through changes in the nodal structure of the molecular orbitals actively involved in the transition. While the S${}_{0}$–S${}_{2}$ transition is also characterized by changes in the methine bridge, the electron density transfers from the benzene ring of subsystem II to subsystem I, thus indicating the pronounced CT character upon excitation. Indeed, the $\Delta \mu (S_{2}$–$S_{0})$ is 5.7 Debye, which is almost 3 times larger than that of the S${}_{0}$–S${}_{1}$ transition. The analysis of differential electron densities depicted in Figure 2b also supports these conclusions. The $\Delta \rho (S_{1}$–$S_{0})$ is mainly localized in subsystem I, while the $\Delta \rho (S_{2}$–$S_{0})$ corresponds to electron density transfer from subsystem II to subsystem I. Therefore, in compound **I**, the S${}_{0}$–S${}_{1}$ transition primarily refers to *intra*-subsystem excitation and is expected to be optically bright, with an oscillator strength being inherited from the GFP chromophore. The S${}_{0}$–S${}_{2}$ transition refers to *inter*-subsystem excitation and, thus, exhibits a low oscillator strength. We will refer to the first and second transitions as of type I and II, respectively.

Applying the “particle in a box” concept to their transition energies, we might expect that both orbitals involved in transitions of type II will be affected by extension of the π-conjugated system upon annulation of the benzene ring, resulting in a red shift. At the same time, local transitions of type I are expected to be affected to a lesser extent; however, since annulation of the benzene ring may efficiently couple two subsystems, it is notoriously harder to predict an impact of various modes of annulation on type I transitions energies. In some cases, this may result in a flip of optically bright and dark states, as it is indeed observed in compound **1A** (see Table 2).

We note that the analysis of electron density redistribution upon various excitations is performed in so-called Franck-Condon points, i.e., ground-state equilibrium geometries of the molecules studied in this work. Since the molecules are designed to be planar and conformationally rigid, we do not expect significant changes in transition dipole moments of the lowest-energy transition calculated at their equilibrium geometries either in S${}_{0}$ or S${}_{1}$. Therefore, if the S${}_{0}$–S${}_{1}$ transition is found to be optically dark at the Franck-Condon point, very low FQYs are also assumed for radiative transitions from S${}_{1}$. In the following, we analyze the impact of various modes of annulation on transition energies and oscillator strengths of the S${}_{0}$–S${}_{1}$ and S${}_{0}$–S${}_{2}$ transitions in compounds from classes **A**, **B**, **C**, and **D**.

### 3.1. Class A

**Figure 3:**
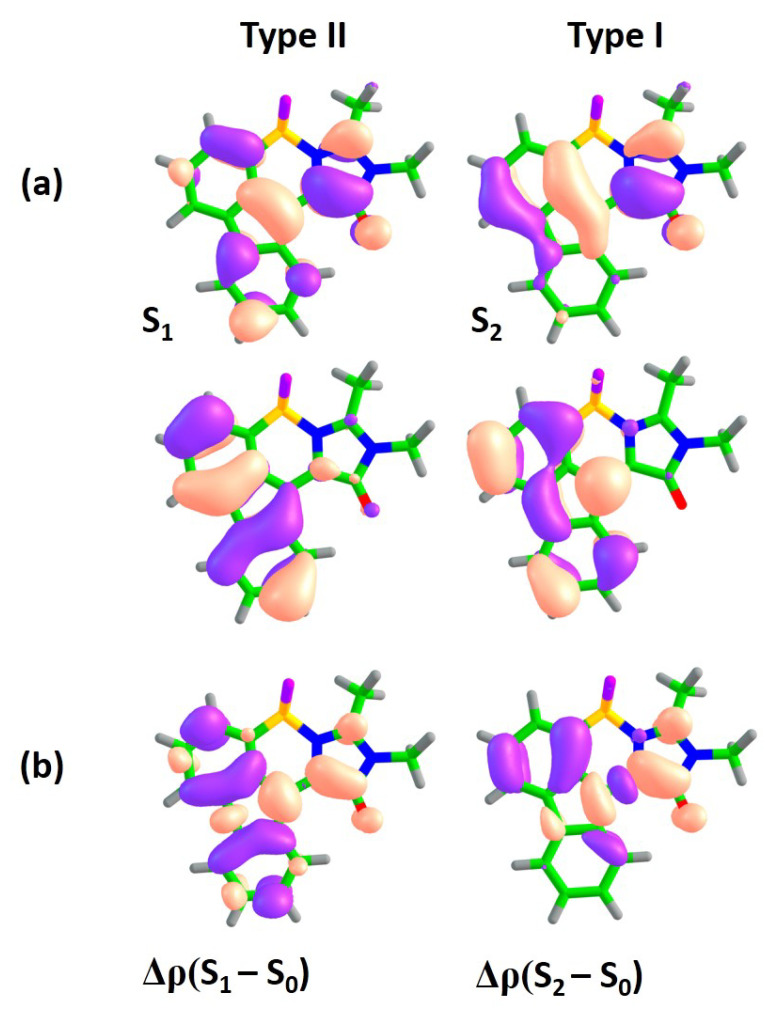
XMCQDPT2 natural orbitals actively involved in the S${}_{0}$–S${}_{1}$ and S${}_{0}$–S${}_{2}$ transitions (**a**) and the corresponding differential electron densities Δρ (**b**) for compound **1A**. For each state, the excitation is directed from the lower orbital to the higher orbital. The color code stands for negative and positive Δρ values depicted in blue and red, respectively.

Compounds of class **A** are derived through a symmetric mode of annulation of compound **1** based on the addition of fluorene-based PAHs to the central methine bridge (Figure 1). As it is discussed above, the experimental photophysical properties of compound **1A** are consistent with the states switching phenomenon. Indeed, Figure 3 shows that the natural orbitals involved in the type II transition become delocalized over fluorene, whereas the electron density redistribution upon the type I transition predominantly occurs in subsystem I, although some coupling to subsystem II is also observed. As a result, the energy of the type I transition is only slightly red shifted from 387 nm in compound **1** to 405 nm in compound **1A**, whereas the type II transition considerably drops in energy from 344 nm to 486 nm (see Table 2). Therefore, the low FQY observed experimentally for compound **1A** can be explained by the states switching and applying the Kasha’s rule.

**Figure 4:**
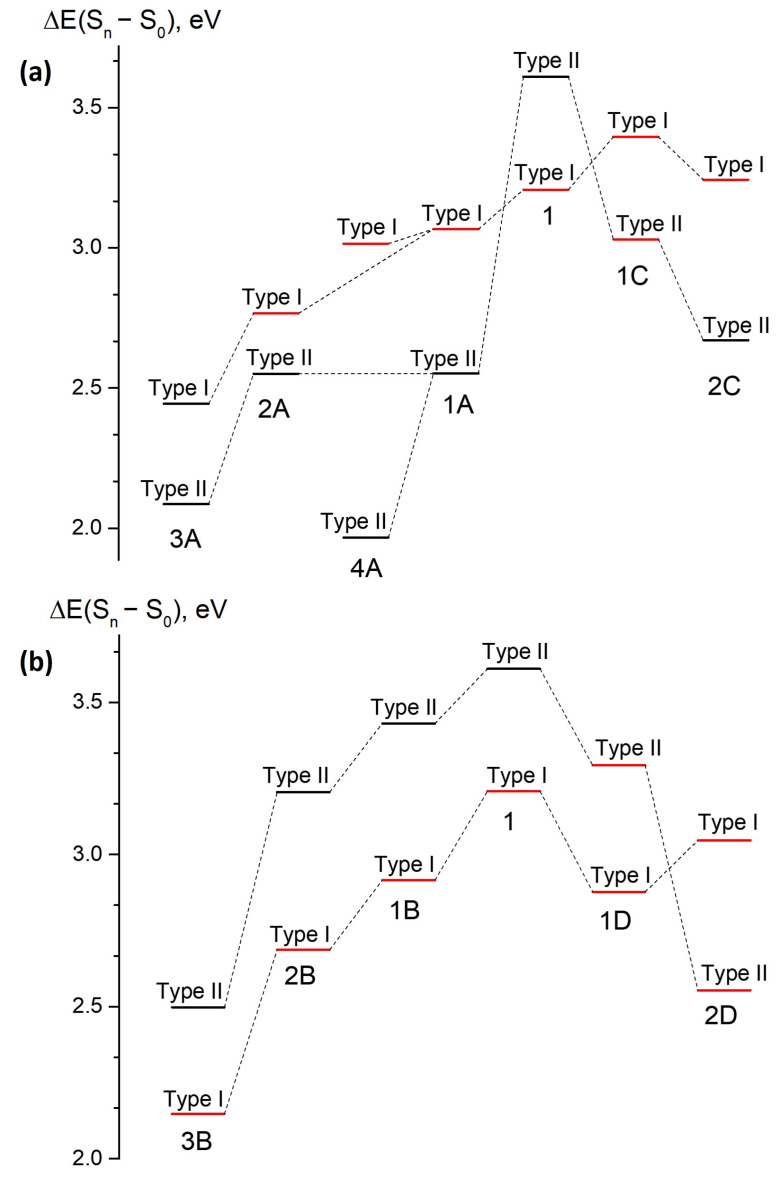
XMCQDPT2 vertical excitation energies for type I and type II transitions in the annulated locked GFP chromopore derivatives obtained through symmetric (**a**) and asymmetric (**b**) modes of annulation. Transitions with relatively high and low oscillator strengths are highlighted in red and black, respectively.

Larger molecules of class **A** demonstrate the same tendency of the inverted order of the optically bright and dark states, as shown in Figure 4 (also see Figures S2–S7 in the Supplementary Materials). Compounds **2A**, **3A**, and **4A** also exhibit relatively high oscillator strengths for transitions to higher-lying S${}_{3}$ and S${}_{4}$ states. Moreover, compound **3A** has a low oscillator strength of 0.08 for both the S${}_{0}$–S${}_{1}$ and S${}_{0}$–S${}_{2}$ transitions. At the same time, the type II transition, which is completely dark in compounds **1A** and **2A**, acquires some intensity in compounds **3A** and **4A**. This indicates mixing of states; hence, the transition intensity becomes diluted over a number of excited states in the large molecules of class **A**. All molecules of class **A** exhibit very low oscillator strengths for the S${}_{0}$–S${}_{1}$ transition, and they are, therefore, expected to have low FQYs, thus making this mode of annulation inappropriate for developing bright red-shifted fluorescent probes.

### 3.2. Class B

**Figure 5:**
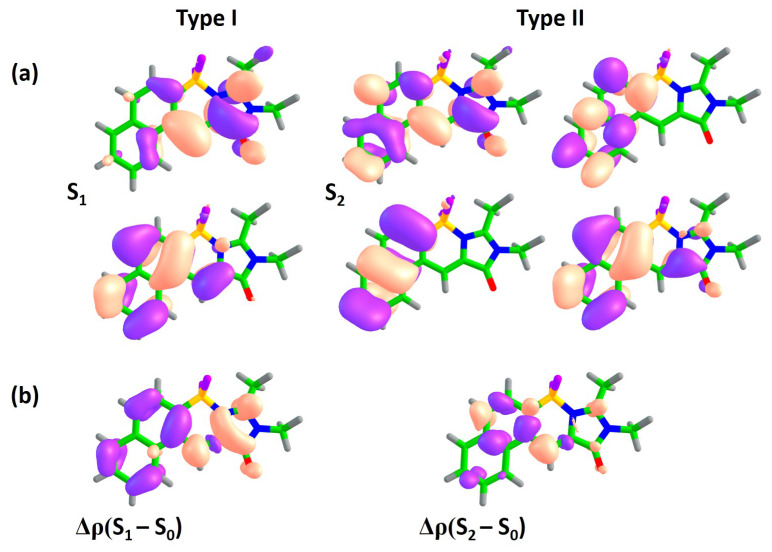
XMCQDPT2 natural orbitals actively involved in the S${}_{0}$–S${}_{1}$ and S${}_{0}$–S${}_{2}$ transitions (**a**) and the corresponding differential electron densities Δρ (**b**) for compound **1B**. For each state, the excitation is directed from the lower orbital to the higher orbital. The color code stands for negative and positive Δρ values depicted in blue and red, respectively.

Molecules from class **B** represent an asymmetric mode of annulation (Figure 1). This class shows an opposite trend with respect to that found for class **A**. Figure 4 shows that no switching of states occurs upon annulation in this case. The S${}_{0}$–S${}_{1}$ transition remains to be of type I for all compounds in this class, and transition energies to both the S${}_{1}$ and S${}_{2}$ states monotonically shifts to longer wavelengths as the molecular size increases. Figure 5 shows natural orbitals involved in type I and type II transitions in compound **1B**, indicating that the two subsystems are efficiently coupled in this case and the annulation affects both transitions. The type I transition also acquires a CT character in addition to the local excitation in subsystem I. Indeed, the $\Delta \mu (S_{1}$–$S_{0})$ becomes as high as 9.4 Debye. At the same time, the type II transition becomes more complex with four actively involved natural orbitals. The local excitation in subsystem II makes a significant contribution to the S${}_{0}$–S${}_{2}$ transition, diminishing its CT character in accordance with a reduced value of $\Delta \mu (S_{2}$–$S_{0})$, which becomes 2.8 Debye.

Larger molecules of this class show the same trend (see Figure 4 and Figures S8–S9 in the Supplementary Materials). For all molecules of class **B**, the S${}_{0}$–S${}_{1}$ transition has the highest oscillator strength, and no other higher-lying states acquire intensity as the molecular size increases. These results are in good agreement with the experimentally observed high FQY for compound **1B**. Subsystems I and II are coupled in class **B** in such a way that the type I transition can fully benefit from the asymmetric mode of annulation, acquiring a significant CT character and a red-shifted excitation energy. This is in contrast to the results for class **A**, where the type I transition is merely affected by the annulation, preserving the local excitation character within subsystem I for compounds **1A** and **2A**. Therefore, the asymmetric mode of annulation in class **B** demonstrates a promising strategy for designing red-shifted fluorophores, reaching a vertical excitation energy of 578 nm in compound **3B** with an appreciable oscillator strength (Table 2).

### 3.3. Class C

**Figure 6:**
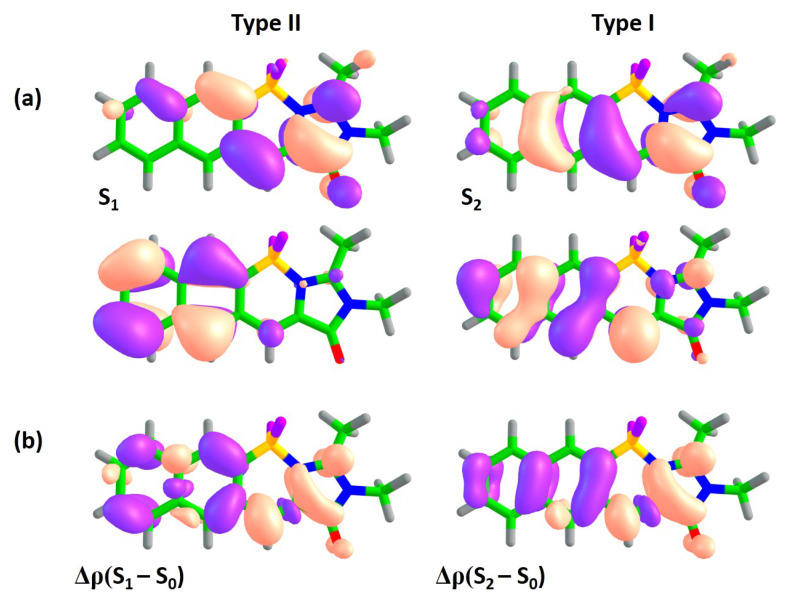
XMCQDPT2 natural orbitals actively involved in the S${}_{0}$–S${}_{1}$ and S${}_{0}$–S${}_{2}$ transitions (**a**) and the corresponding differential electron densities Δρ (**b**) for compound **1C**. For each state, the excitation is directed from the lower orbital to the higher orbital. The color code stands for negative and positive Δρ values depicted in blue and red, respectively.

Molecules of class **C** represent another symmetric mode of annulation, which is based on the extension of the benzene ring of subsystem II to naphthalene and anthracene (Figure 1). The results for this class resemble those observed for ‘symmetric’ class **A**. State switching occurs upon annulation, as it is shown in Figure 4 and Figure 6 (also see Figures S10 and S11 in the Supplementary Materials).

While the type II transition in compound **1C** demonstrates a relatively high oscillator strength of 0.14, it is much lower than that of the type I transition (0.69). The oscillator strengths for both transitions decrease in compound **2C**, while the higher-lying S${}_{0}$–S${}_{3}$ transition acquires the highest oscillator strength. This mode of annulation will most likely result in significant decrease of FQYs compared to that of compound **1**.

### 3.4. Class D

**Figure 7:**
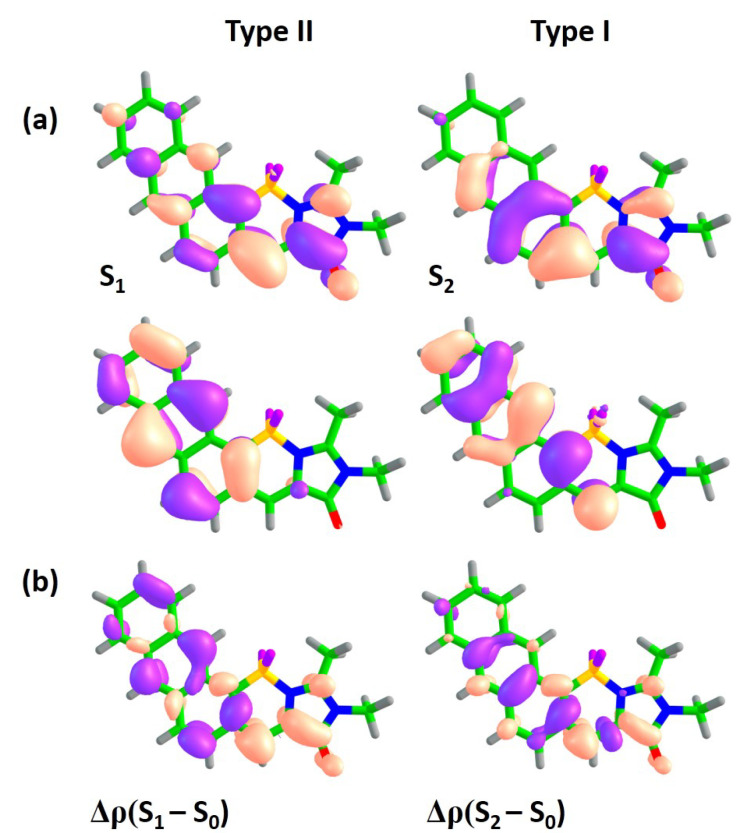
XMCQDPT2 natural orbitals actively involved in the S${}_{0}$–S${}_{1}$ and S${}_{0}$–S${}_{2}$ transitions (**a**) and the corresponding differential electron densities Δρ (**b**) for compound **2D**. For each state, the excitation is directed from the lower orbital to the higher orbital. The color code stands for negative and positive Δρ values depicted in blue and red, respectively.

Class **D** represents another asymmetric mode of annulation. The results for this class are similar to those of ‘asymmetric’ class **B**. As could be seen from Figure 4 and Figure 7, class **D** demonstrates switching of states upon annulation; however, it only occurs for compound **2D**, while it is not observed for molecule **1D** (also see Figures S12 and S13 in the Supplementary Materials). The two low-lying transitions have relatively large oscillator strengths in compound **1D**. Based on the analysis of the electron density redistribution, the lowest-energy transition can still be referred to as type I in compound **1D**, although the corresponding oscillator strength appears to be smaller compared to that of the second transition. This indicates a pronounced coupling between subsystems I and II, which affects both transitions in this case. The lowest-energy type I transition is also characterized by a large CT character, which is reflected in the large $\Delta \mu (S_{1}$–$S_{0})$ of 9.9 Debye. Upon further increase of the molecular size, the order of states changes and the most intense transition also shifts to higher-lying states (see Table 2).

At the same time, the oscillator strength for the altered type II transition is relatively high and equal to 0.18 for compound **2D**, which is in contrast to those observed for all other classes. Therefore, class **D** can in principle be used for developing red-shifted fluorophores with an altered pattern of excited states. As the molecular size increases, we expect that the oscillator strength of the S${}_{0}$–S${}_{1}$ transition will be reduced, although it could still be relatively high for several larger molecules of this class.

Further studies of even larger compounds from classes **B** and **D**, as well as those obtained from **1** by combining various modes of annulation presented in this work, could be of interest in case of their synthetic accessibility and possible biological acceptability, considering the molecular size limits imposed on chromophores for particular bioimaging applications [38]. We note that annulation, although red shifting absorption and emission, inevitably demonstrates an overall decrease in oscillator strengths of the S${}_{0}$–S${}_{1}$ transition due to its *inter*-subsystem excitation character. Among the four classes with various modes of annulation, class B is the most promising for developing relatively bright red-shifted molecular emitters with an absorption wavelength of ∼600 nm. This class exhibits an efficient coupling between subsystems I and II, which affects both transitions; thus, it avoids the switching between optically dark and bright states. In future studies, further tuning can be gained by introducing electron-withdrawing or electron-donating groups, for example, to the central atom of the methine bridge [21].

## 4. Conclusions

We demonstrate that high-level ab initio XMCQDPT2 calculations can be used to predict the most promising ways of polycyclic modifications of the BF_2_-locked GFP chromophore derivative to obtain fluorophores with red-shifted absorption and emission. We consider four novel classes with symmetric and asymmetric modes of annulation and show that they provide remarkably different patterns of coupling between the electronic structures of the GFP chromophore’s core and its polycyclic extension. The symmetric modes of annulation in classes **A** and **C** result in a flip of optically bright and dark states, and the lowest-lying excited state turns out to be completely dark, thus preventing radiative transitions from S${}_{1}$. The presence of the completely dark lowest-lying excited state is supported by the experimentally measured extremely low fluorescence quantum yield of newly synthesized compound **1A**. Therefore, the switching of states, reported in the present work, should be taken into account when designing fluorophores with red-shifted absorption and emission based on extension of their π-conjugated system through rigid polycyclic modifications. At the same time, we reveal that the asymmetric modes of annulation in classes **B** and **D** turn out to be promising strategies to extend the π-conjugation of the locked GFP chromophore derivative. The largest molecule from class **B** exhibits the most red-shifted absorption at ∼600 nm, which is characterized by a relatively high oscillator strength. Our approach can further be expanded to the analysis of alternative modes of annulation or to other simple reference molecules, thus making valuable contributions to the computer-aided design of bright red-shifted molecular emitters for bioimaging applications.

## Figures and Tables

**Table 1:** Experimental photophysical properties of compounds **1**, **1A**, and **1B** in various solvents.

Solvent		1A ${}^{c}$	1 [14]	1B [15]
Water	Abs ${}^{a}$	– ${}^{d}$	368 (–)	427 (–) ${}^{e}$
	Em ${}^{b}$	– ${}^{d}$	430 (∼10)	532 (65)
MeOH	Abs ${}^{a}$	415 (11.5)	–	–
	Em ${}^{b}$	∼470 (<0.2)	–	–
EtOH	Abs ${}^{a}$	– ${}^{d}$	365 (–)	436 (–) ${}^{e}$
	Em ${}^{b}$	– ${}^{d}$	426 (∼20)	510 (30)
CH_3_CN	Abs ${}^{a}$	414 (13)	360 (–)	425 (19)
	Em ${}^{b}$	∼470 (<0.2)	420 (55)	497 (86)
EtOAc	Abs ${}^{a}$	416 (13.5)	362 (–)	428 (19.5)
	Em ${}^{b}$	∼470 (<0.2)	421 (63)	484 (95)
Dioxane	Abs ${}^{a}$	419 (12.5)	365 (–)	435 (20)
	Em ${}^{b}$	∼470 (<0.2)	422 (78)	483 (98)

${}^{a}$ Peak maximum in nm (extinction coefficient in (mM·cm)${}^{-1}$). ${}^{b}$ Peak maximum in nm (fluorescence quantum yield in %). ${}^{c}$ Synthesized in this work. ${}^{d}$ Not registered. ${}^{e}$ Not soluble enough.

**Table 2:** Calculated photophysical properties for the $S_{0}$–$S_{n}$ transitions in compound **1** and its derivatives from classes **A**, **B**, **C**, and **D** in the gas phase.

Compound	State	Oscillator Strength	$\lambda_{\mathrm{excitation}}$, nm	$\Delta \mu (S_{n}-S_{0})$, D
**1**	S_1_	0.58	387	2.2
	S_2_	0.01	344	5.7
**1A**	S_1_	2·10${}^{-5}$	486	6.0
	S_2_	0.32	405	6.5
**2A**	S_1_	3·10${}^{-3}$	486	5.5
	S_2_	0.35	448	6.8
	S_3_	0.13	394	3.2
**3A**	S_1_	0.08	595	8.4
	S_2_	0.08	508	5.5
	S_3_	0.11	408	3.1
	S_4_	0.14	391	2.8
**4A**	S_1_	0.03	631	7.7
	S_2_	0.16	411	3.4
	S_3_	0.02	386	2.3
	S_4_	0.14	375	5.9
**1B**	S_1_	0.45	426	9.4
	S_2_	0.01	362	2.8
**2B**	S_1_	0.35	462	10.6
	S_2_	8·10${}^{-3}$	387	1.5
**3B**	S_1_	0.17	578	9.6
	S_2_	4·10${}^{-3}$	497	1.2
**1C**	S_1_	0.14	409	7.4
	S_2_	0.69	365	9.7
**2C**	S_1_	0.09	465	5.4
	S_2_	0.59	383	5.8
	S_3_	0.63	343	4.3
**1D**	S_1_	0.24	431	9.9
	S_2_	0.33	377	6.3
**2D**	S_1_	0.18	486	9.0
	S_2_	0.31	407	3.6
	S_3_	0.40	340	2.1
	S_4_	0.26	336	2.3

## Data Availability

Data for compound **1A** are available from the authors.

## Supplementary Materials

The following are available online at https://www.mdpi.com/article/10.3390/ijms222413645/s1.
