# Supplementary material for: Designing Red-Shifted Molecular Emitters Based on the Annulated Locked GFP Chromophore Derivatives
Source: Int J Mol Sci. 2021 Dec 20;22(24):13645. doi: 10.3390/ijms222413645 (PMC8703576; doi:10.3390/ijms222413645)
Supplement: Supplementary file 1 [file ijms-22-13645-s001.zip › ijms-1514880-supplementary.pdf]

Supplementary Materials: Designing Red-Shifted Molecular Emitters Based on the Annulated Locked GFP Chromophore Derivatives

Gregory D. Sinenko [1], Dilara A. Farkhutdinova [1], Ivan N. Myasnyanko [2,3], Nadezhda S. Baleeva [2,3], Mikhail S. Baranov [2,3], and Anastasia V. Bochenkova [1,*]

Contents

1. Experimental absorption and emission spectra of compound 1A 2
2. Eigenvectors of the XMCQDPT2 effective Hamiltonian 3
3. Natural orbitals and differential electron densities 4
4. Cartesian coordinates of optimized structures 10

1. Experimental absorption and emission spectra of compound 1A

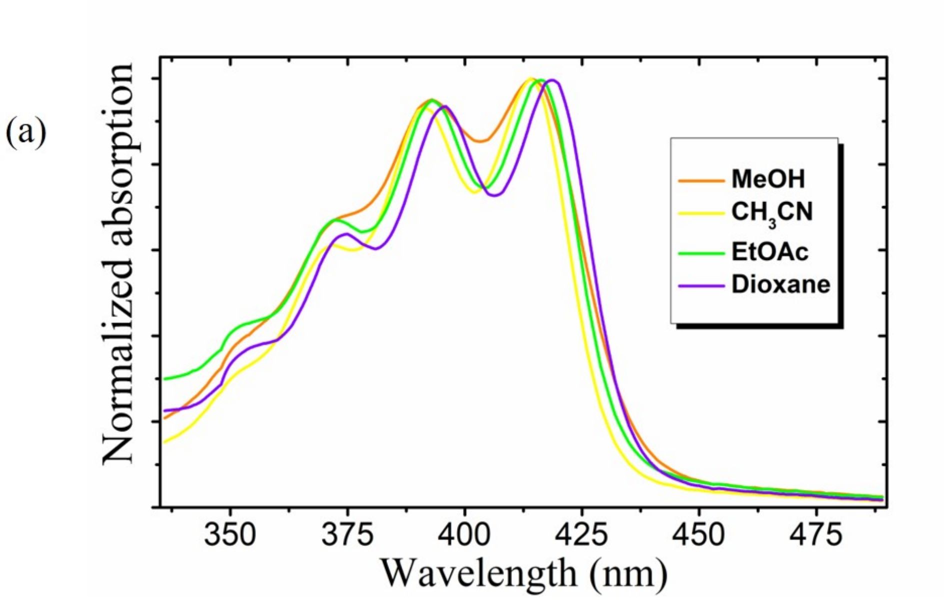

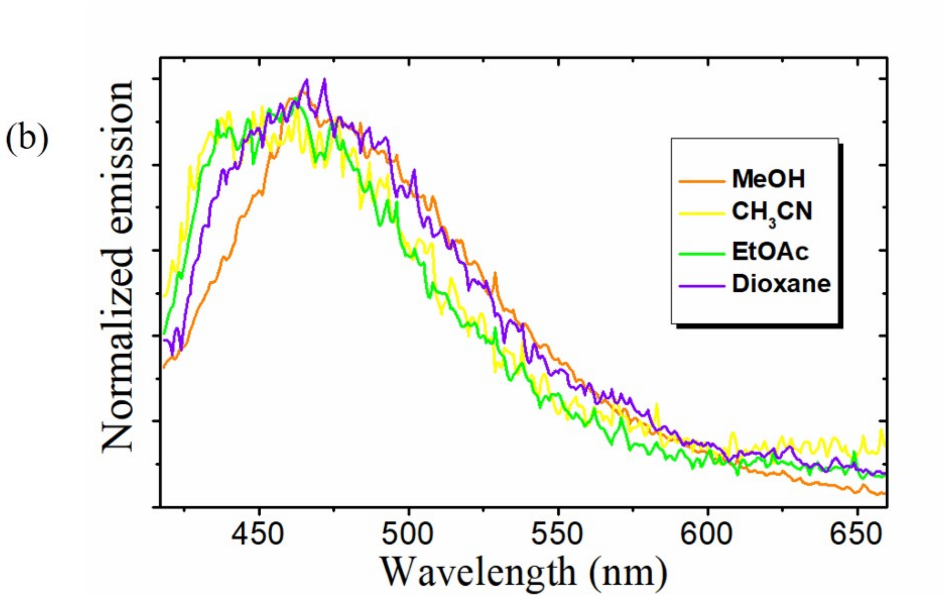

Figure S1. Experimental absorption (a) and emission (b) spectra of compound **1A** in various solvents.

2. Eigenvectors of the XMCQDPT2 effective Hamiltonian

Table S1: Eigenvectors of the XMCQDPT2 effective Hamiltonian for compound **1A**. The perturbed CASSCF states are obtained after diagonalization of the effective Hamiltonian and represented by the linear combinations of the initial CASSCF reference states.

Perturbed → Reference ↓	1	2	3	4	5	6	7
1	-0.9996	0.0025	0.0254	0.0031	0.0081	-0.0029	0.0019
2	-0.0026	-0.9836	-0.0198	0.0797	0.0072	-0.1164	-0.1108
3	0.0143	-0.0575	0.5949	-0.7674	0.2117	-0.0579	-0.0737
4	-0.0192	-0.0308	-0.4756	-0.5491	-0.6829	-0.0669	-0.0101
5	0.0123	0.0062	0.6400	0.3044	-0.6808	-0.1248	0.1360
6	-0.0021	-0.0142	0.0951	0.0378	-0.1585	0.7371	-0.6488
7	0.0008	0.1677	-0.0156	0.0953	0.0010	-0.6479	-0.7367

3. Natural orbitals and differential electron densities

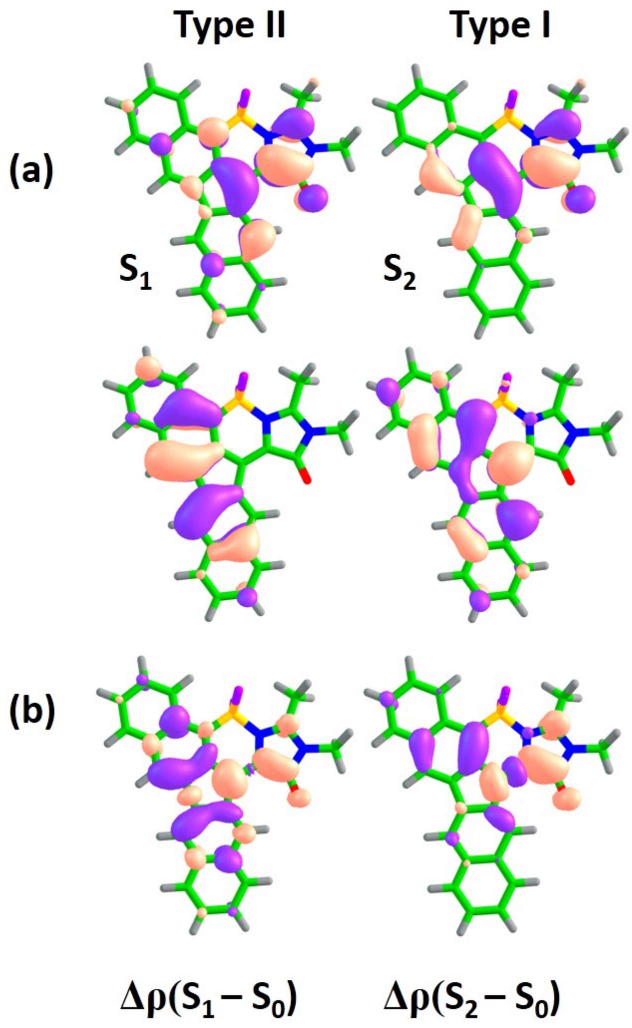

Figure S2. (a) XMCQDPT2 natural orbitals actively involved in the S_0-S_1 and S_0-S_2 transitions for compound **2A**. For each state, the excitation is directed from the lower orbital to the higher orbital. (b) XMCQDPT2 $\Delta\rho(S_1 - S_0)$ and $\Delta\rho(S_2 - S_0)$ for compound **2A**. The blue color designates the area with a negative $\Delta\rho$, while the red color corresponds to positive $\Delta\rho$ values.

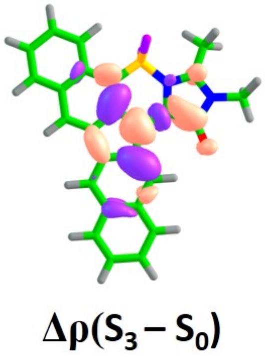

Figure S3. XMCQDPT2 $\Delta\rho(S_3 - S_0)$ for compound **2A**. The blue color designates the area with a negative $\Delta\rho$, while the red color corresponds to positive $\Delta\rho$ values.

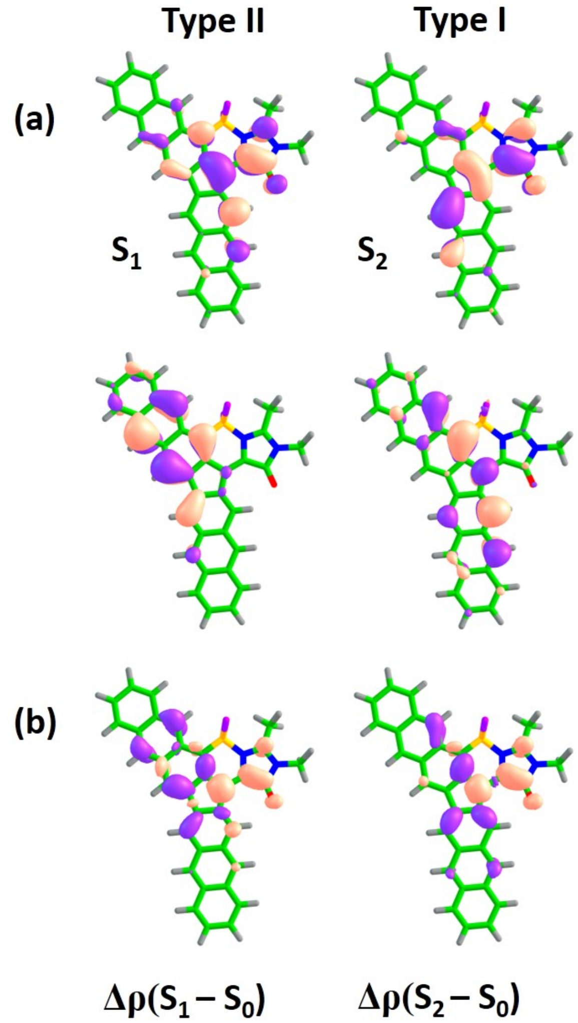

Figure S4. (a) XMCQDPT2 natural orbitals actively involved in the S_0-S_1 and S_0-S_2 transitions for compound **3A**. For each state, the excitation is directed from the lower orbital to the higher orbital. (b) XMCQDPT2 $\Delta\rho(S_1 - S_0)$ and $\Delta\rho(S_2 - S_0)$ for compound **3A**. The blue color designates the area with a negative $\Delta\rho$, while the red color corresponds to positive $\Delta\rho$ values.

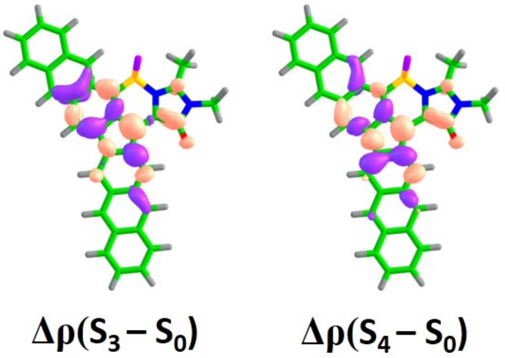

Figure S5. XMCQDPT2 $\Delta\rho(S_3 - S_0)$ and $\Delta\rho(S_4 - S_0)$ for compound **3A**. The blue color designates the area with a negative $\Delta\rho$, while the red color corresponds to positive $\Delta\rho$ values.

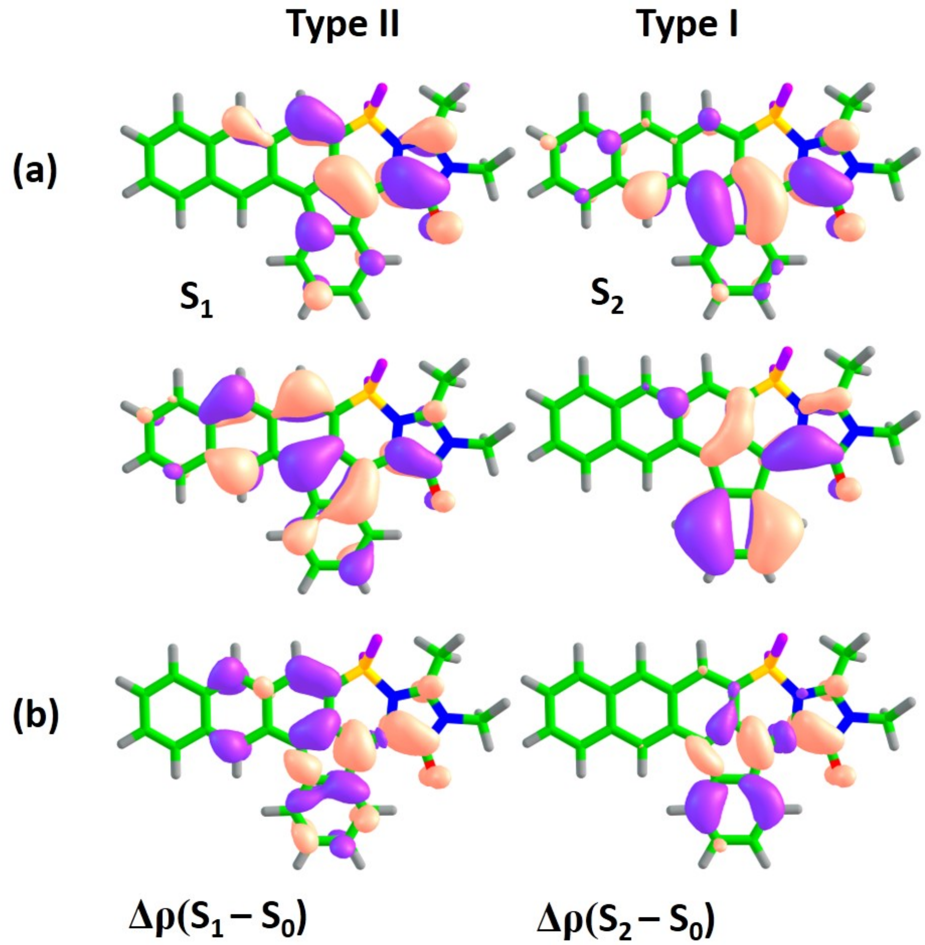

Figure S6. (a) XMCQDPT2 natural orbitals actively involved in the S_0-S_1 and S_0-S_2 transitions for compound **4A**. For each state, the excitation is directed from the lower orbital to the higher orbital. (b) XMCQDPT2 $\Delta\rho(S_1 - S_0)$ and $\Delta\rho(S_2 - S_0)$ for compound **4A**. The blue color designates the area with a negative $\Delta\rho$, while the red color corresponds to positive $\Delta\rho$ values.

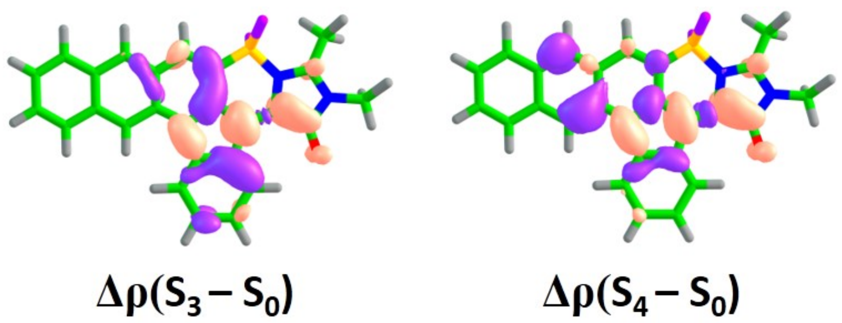

Figure S7. XMCQDPT2 $\Delta\rho(S_3 - S_0)$ and $\Delta\rho(S_4 - S_0)$ for compound **4A**. The blue color designates the area with a negative $\Delta\rho$, while the red color corresponds to positive $\Delta\rho$ values.

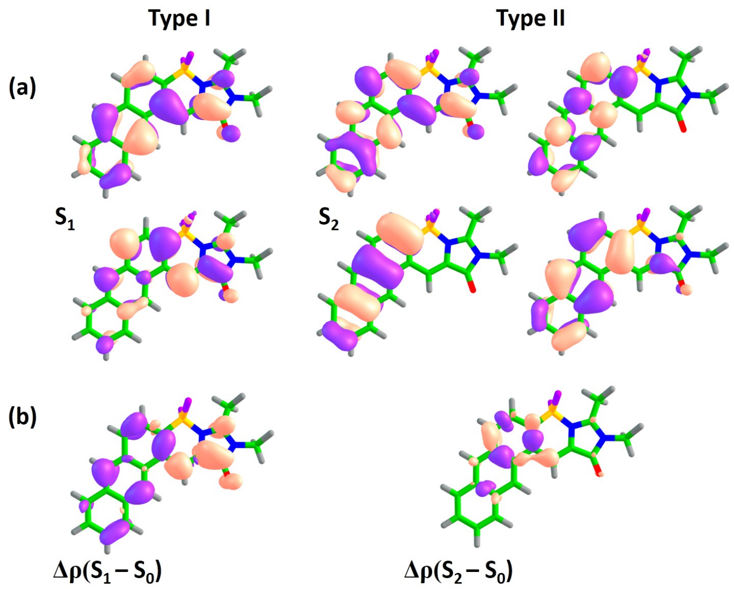

Figure S8. (a) XMCQDPT2 natural orbitals actively involved in the S_0-S_1 and S_0-S_2 transitions for compound **2B**. For each state, the excitation is directed from the lower orbital to the higher orbital. (b) XMCQDPT2 $\Delta\rho(S_1 - S_0)$ and $\Delta\rho(S_2 - S_0)$ for compound **2B**. The blue color designates the area with a negative $\Delta\rho$, while the red color corresponds to positive $\Delta\rho$ values.

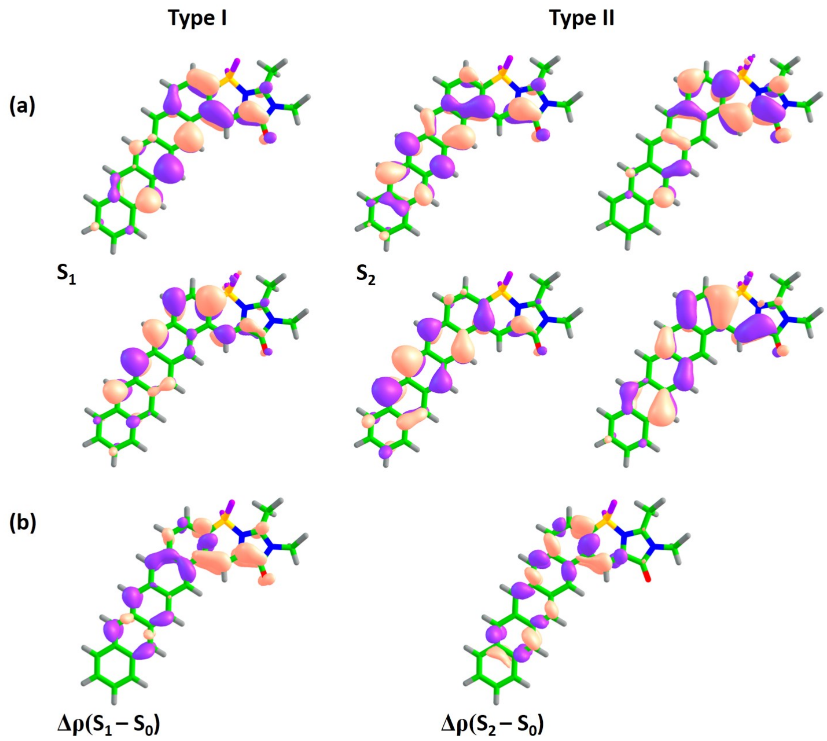

Figure S9. (a) XMCQDPT2 natural orbitals actively involved in the S_0-S_1 and S_0-S_2 transitions for compound **3B**. For each state, the excitation is directed from the lower orbital to the higher orbital. (b) XMCQDPT2 $\Delta\rho(S_1 - S_0)$ and $\Delta\rho(S_2 - S_0)$ for compound **3B**. The blue color designates the area with a negative $\Delta\rho$, while the red color corresponds to positive $\Delta\rho$ values.

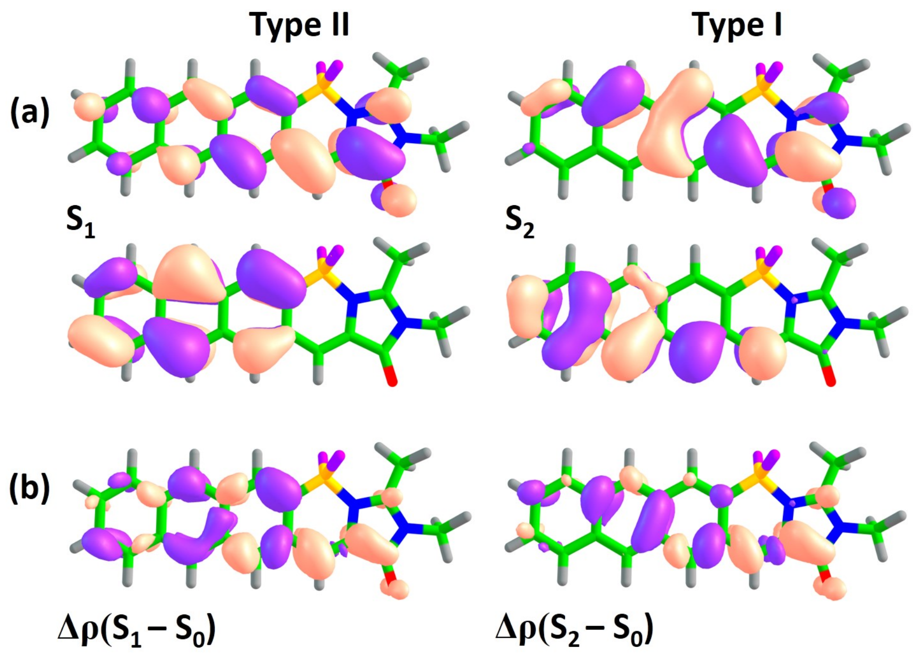

Figure S10. (a) XMCQDPT2 natural orbitals actively involved in the S_0-S_1 and S_0-S_2 transitions for compound **2C**. For each state, the excitation is directed from the lower orbital to the higher orbital. (b) XMCQDPT2 $\Delta\rho(S_1 - S_0)$ and $\Delta\rho(S_2 - S_0)$ for compound **2C**. The blue color designates the area with a negative $\Delta\rho$, while the red color corresponds to positive $\Delta\rho$ values.

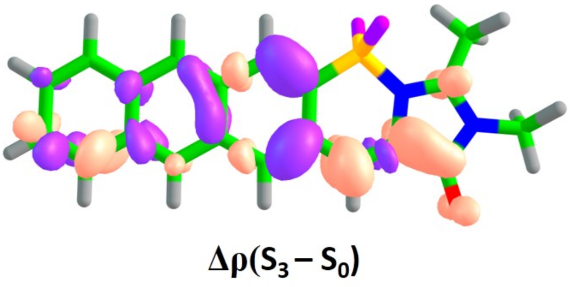

Figure S11. XMCQDPT2 $\Delta\rho(S_3 - S_0)$ for compound **2C**. The blue color designates the area with a negative $\Delta\rho$, while the red color corresponds to positive $\Delta\rho$ values.

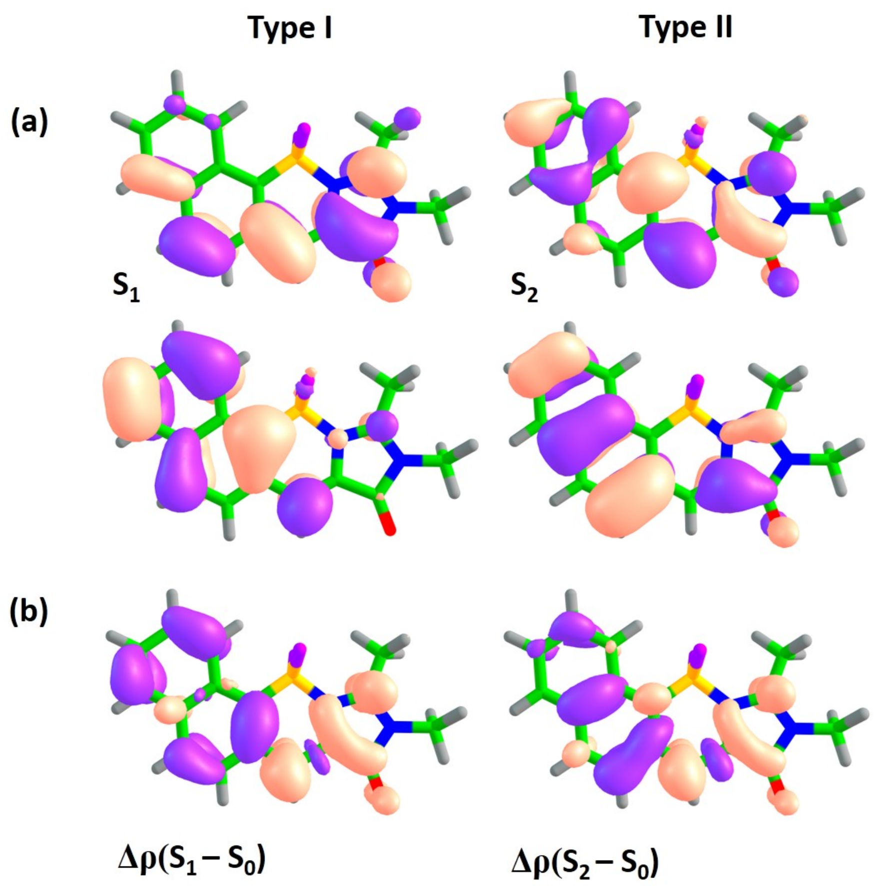

Figure S12. (a) XMCQDPT2 natural orbitals actively involved in the S_0-S_1 and S_0-S_2 transitions for compound **1D**. For each state, the excitation is directed from the lower orbital to the higher orbital. (b) XMCQDPT2 $\Delta\rho(S_1 - S_0)$ and $\Delta\rho(S_2 - S_0)$ for compound **1D**. The blue color designates the area with a negative $\Delta\rho$, while the red color corresponds to positive $\Delta\rho$ values.

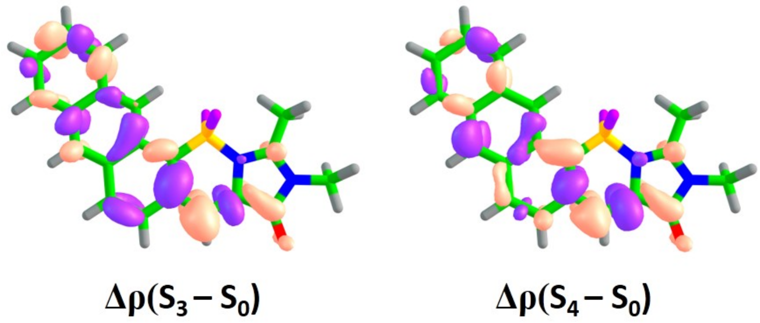

Figure S13. XMCQDPT2 $\Delta\rho(S_3 - S_0)$ and $\Delta\rho(S_4 - S_0)$ for compound **2D**. The blue color designates the area with a negative $\Delta\rho$, while the red color corresponds to positive $\Delta\rho$ values.

4. Cartesian coordinates of optimized structures

Compound **1**
Energy = -868.3937865049 Hartree
C -5.895315092 -1.074105304 -5.497953848
C -7.012642893 -1.399964963 -4.705686712
C -4.672889950 -0.784974183 -4.880980844
H -7.973403747 -1.622431171 -5.183214564
C -6.897660463 -1.441469219 -3.303495055
C -4.564035536 -0.827055331 -3.467705980
H -7.777611391 -1.695207220 -2.701377974
C -5.682300511 -1.166359881 -2.647363095
H -5.981657410 -1.042591631 -6.589055033
C -3.293290895 -0.486672990 -2.855626122
N -4.160188693 -0.699474907 -0.593205311
C -3.686719018 -0.558115479 0.636242040
C -3.125606290 -0.423959454 -1.498343364
C -1.918853349 -0.071341327 -0.726392684
N -2.367086922 -0.183543545 0.621392985
B -5.612031397 -1.285578081 -1.038981412
F -5.628379284 -2.612406957 -0.566876125
F -6.580013085 -0.507842565 -0.371436705
C -1.516167062 0.078570278 1.774805374
H -1.875238693 0.955992984 2.335421997
H -1.478517446 -0.800949419 2.435806483
H -0.511221111 0.284827767 1.377669955
C -4.458769207 -0.803163412 1.883564859
H -5.529050490 -0.825690967 1.643231724
H -4.161306435 -1.772425249 2.320231902
H -4.253748205 -0.011145792 2.621450604
O -0.784488160 0.251062036 -1.069106117
H -2.422755940 -0.248122400 -3.479795041
H -3.794683260 -0.521086283 -5.482176535

Compound **1A**
Energy = -1096.7995279245 Hartree
C -2.756060148 -0.084171159 -6.497820503
C -3.298525784 -0.307289564 -5.227519301
C -1.418631374 0.352084813 -6.600019379
H -0.982188583 0.529838828 -7.588874723
C -2.501256355 -0.092001606 -4.056799119
C -0.637576756 0.562309572 -5.447245264
H 0.398386394 0.901085383 -5.551297024
C -1.167457778 0.343689554 -4.160749529
H -0.566461319 0.505332643 -3.262480222
H -3.356908968 -0.244937639 -7.399710607
C -5.800066354 -1.108454020 -5.513071385
C -6.935096242 -1.505155157 -4.768761635
C -4.642682725 -0.757701408 -4.809719474
H -7.851532073 -1.782382354 -5.301974818
C -6.916124566 -1.553806933 -3.359163142
C -4.653574953 -0.815461387 -3.391449050
H -7.821590416 -1.868222205 -2.827596063
C -5.760847484 -1.207799374 -2.618525190
H -5.837187794 -1.079387938 -6.607912317

C -3.340775209 -0.404391991 -2.892051756
N -4.136214260 -0.722176196 -0.638224853
C -3.662579356 -0.627428913 0.598403092
C -3.109839306 -0.369572318 -1.532257022
C -1.914439900 -0.025202423 -0.738144071
N -2.352948105 -0.222304484 0.597653348
B -5.660445309 -1.248645400 -1.014719978
F -5.736931889 -2.533787616 -0.449737551
F -6.531951645 -0.359807959 -0.355949636
C -1.498998226 -0.003914616 1.758407435
H -1.863785634 0.844195541 2.359002688
H -1.451550277 -0.912230335 2.378308593
H -0.497875309 0.229415152 1.366928443
C -4.420701464 -0.946541290 1.838401197
H -5.495801841 -0.875521531 1.632820544
H -4.185718438 -1.975013569 2.163224770
H -4.142816256 -0.250082896 2.644719268
O -0.782987090 0.345160505 -1.052863356

Compound **2A**
Energy = -1402.1239179094 Hartree
C -2.780714430 -0.123069796 -6.498504865
C -3.320120347 -0.343827491 -5.241619840
C -1.421457155 0.301717948 -6.624051390
C -2.523122487 -0.116642647 -4.053300727
C -0.627567723 0.521309847 -5.434786736
C -1.195422052 0.297249387 -4.144785654
H -0.599430699 0.461760812 -3.241787015
H -3.377038073 -0.285290157 -7.405200889
C -5.807308692 -1.120637194 -5.523272458
C -6.979379358 -1.505038081 -4.795769192
C -4.665858268 -0.767513615 -4.823067802
C -6.961114000 -1.553572012 -3.345051498
C -4.673947427 -0.829863903 -3.388944335
C -5.774746068 -1.200980923 -2.615492652
H -5.842654332 -1.094947814 -6.619985230
C -3.362212544 -0.423971406 -2.892249861
N -4.146087970 -0.723522661 -0.634826052
C -3.671136806 -0.623312940 0.603404785
C -3.122625001 -0.378794192 -1.530897584
C -1.927601627 -0.033534253 -0.746691118
N -2.362842732 -0.220140890 0.593395001
B -5.656694168 -1.252949504 -1.008161071
F -5.730572476 -2.546873062 -0.458781836
F -6.539752671 -0.381087280 -0.336147729
C -1.503110660 0.003093990 1.748617619
H -1.865791465 0.852842552 2.348220606
H -1.451359602 -0.902908232 2.371664356
H -0.504512312 0.236176130 1.350557061
C -4.424786231 -0.937680709 1.847492213
H -5.500818286 -0.876501998 1.643932370
H -4.181066901 -1.960744993 2.183126481
H -4.152041121 -0.230978669 2.646857261
O -0.793040547 0.332530049 -1.064591479
C -8.174640736 -1.863118366 -5.483434173

C -9.316585505 -2.242568228 -4.785771442
H -10.226876857 -2.512718120 -5.331902992
C -9.303112554 -2.280864536 -3.365571975
H -10.206516331 -2.576969340 -2.821721734
C -8.153214289 -1.940913455 -2.660982678
H -8.144929648 -1.964591623 -1.567503599
H -8.181632808 -1.829750680 -6.579623163
C 0.496278642 0.950767054 -8.003436845
C -0.826233129 0.531557140 -7.897514007
C 1.277210442 1.161311897 -6.834314023
H -1.428755514 0.368729558 -8.798964998
H 2.317462505 1.490879330 -6.926999693
C 0.725945889 0.948664672 -5.575708641
H 1.322233114 1.109425830 -4.670085786
H 0.937610585 1.120875279 -8.991292111

Compound **3A**
Energy = -1707.4297036717 Hartree
C -2.762934779 -0.106412298 -6.478934938
C -3.304584314 -0.333259309 -5.227268815
C -1.396667686 0.311331642 -6.602747539
C -2.509410509 -0.107262553 -4.028457555
C -0.603240480 0.530093557 -5.400175552
C -1.181831817 0.298838422 -4.111253210
H -0.590780114 0.460084748 -3.204137278
H -3.356768169 -0.264718012 -7.388209860
C -5.788340726 -1.098387355 -5.516424662
C -6.971271219 -1.474083814 -4.793624884
C -4.651287324 -0.746828685 -4.813743224
C -6.952844887 -1.531203639 -3.332624047
C -4.659596525 -0.818739804 -3.372924170
C -5.760225026 -1.184237251 -2.601920428
H -5.820602738 -1.068957701 -6.613358960
C -3.350284076 -0.417530524 -2.872374614
N -4.137053070 -0.718885880 -0.615540197
C -3.665319759 -0.617893220 0.625254591
C -3.112620236 -0.373367183 -1.508409265
C -1.921116025 -0.026200444 -0.723702797
N -2.358870220 -0.211513150 0.616351650
B -5.638234812 -1.262380287 -0.995322271
F -5.694317329 -2.568204226 -0.472605576
F -6.537175383 -0.418063663 -0.307857439
C -1.501127405 0.013637594 1.772484133
H -1.867907413 0.861018826 2.372973288
H -1.446185374 -0.892933509 2.394519991
H -0.503185754 0.251273282 1.375433339
C -4.419091985 -0.937049512 1.868062214
H -5.495119813 -0.892034072 1.660558058
H -4.161538709 -1.954492136 2.210709326
H -4.159889339 -0.222314255 2.664985911
O -0.785085866 0.341395387 -1.038946875
C -8.151631801 -1.821410722 -5.479703115
C -8.134524051 -1.897573419 -2.651970774
H -8.130471312 -1.917990320 -1.556960076
H -8.160359792 -1.785163803 -6.577318978

C -0.805979544 0.541561811 -7.860201161
C 1.328038196 1.172205624 -6.787132874
H -1.403171451 0.382747413 -8.767754902
C 0.738791320 0.946235305 -5.525029378
H 1.332295242 1.103701109 -4.615164566
C -10.530566888 -2.547906860 -5.487719934
C -11.669567049 -2.914175314 -4.788266316
C -9.327136390 -2.198929604 -4.795745663
H -12.583782967 -3.178004086 -5.330800929
C -11.658391547 -2.950505485 -3.359687869
C -9.314409020 -2.240370427 -3.345849891
H -12.565527428 -3.241179340 -2.818908316
C -10.511067610 -2.620812688 -2.656295756
H -10.499562969 -2.648588868 -1.560510942
H -10.536993213 -2.518468760 -6.583949297
C 1.142171620 1.192269011 -9.261842494
C 0.536520089 0.961672156 -7.985450155
C 2.461344917 1.606198696 -9.356732064
H 2.911120502 1.778040441 -10.340436522
C 3.240552363 1.811088301 -8.176674736
H 4.282109519 2.137983499 -8.265792355
C 2.688465691 1.599084174 -6.923682576
H 3.285315957 1.756120158 -6.017599261
H 0.542996900 1.034285727 -10.166453436

Compound **4A**
Energy = -1402.1032527551 Hartree
C -2.632366346 -0.127392062 -6.486140854
C -3.238188501 -0.324213114 -5.236491777
C -1.295062308 0.323170742 -6.546409324
H -0.828688093 0.482203367 -7.524663935
C -2.464916555 -0.100490110 -4.047656664
C -0.555657869 0.556655015 -5.375608481
H 0.479895484 0.905042553 -5.445314349
C -1.136103240 0.349122733 -4.107773942
H -0.570246099 0.520252694 -3.189277724
H -3.170675397 -0.306698468 -7.418880995
C -5.768474302 -1.126152365 -5.567019716
C -6.923640781 -1.576668063 -4.786930488
C -4.585181274 -0.800465324 -4.833652660
C -6.876305514 -1.599383455 -3.352253066
C -4.624682550 -0.827817491 -3.423407330
H -7.775779479 -1.933005874 -2.818485469
C -5.737582415 -1.242402665 -2.636384362
C -3.329821621 -0.408531224 -2.900834849
N -4.144352615 -0.718062166 -0.646379080
C -3.685394404 -0.607470069 0.594031302
C -3.115569822 -0.362239167 -1.535427724
C -1.931693732 0.004035426 -0.729112546
N -2.381219116 -0.186809885 0.602947063
B -5.652956992 -1.266673343 -1.032360335
F -5.719231346 -2.547244566 -0.454620899
F -6.544022266 -0.384214783 -0.390508307
C -1.541421707 0.052029646 1.770104612
H -1.921899455 0.901640860 2.358695491

H -1.490613035 -0.849963157 2.398856872
H -0.539007097 0.292830373 1.386682412
C -4.453798670 -0.926716529 1.827383106
H -5.527051918 -0.855148071 1.611935996
H -4.223278230 -1.956217358 2.152227267
H -4.182281793 -0.231471488 2.636777596
O -0.800698884 0.386711444 -1.030949206
C -7.058355352 -1.466280258 -7.642434194
C -8.203027883 -1.908567568 -6.870236675
C -5.868032256 -1.107199934 -6.975866910
H -5.024442287 -0.768170817 -7.581460411
C -8.107476583 -1.934025539 -5.463262742
H -8.974671746 -2.259956637 -4.874189269
C -8.337192553 -1.799238467 -9.705070192
C -9.467955815 -2.228095472 -8.942641482
C -7.162079386 -1.430634484 -9.071151043
H -10.391087725 -2.515704373 -9.457141651
H -6.294468028 -1.101174031 -9.655344428
C -9.402635079 -2.277799119 -7.559662783
H -10.268497653 -2.606259618 -6.972677427
H -8.402071066 -1.765031106 -10.797913440

Compound **1B**
Energy = -1021.0501699111 Hartree

C -5.824360825 -1.057013190 -5.517416777
C -6.944563463 -1.377507505 -4.696637396
C -4.557725872 -0.754203246 -4.898703149
H -7.905101986 -1.596605636 -5.179185939
C -6.824274185 -1.416193170 -3.311975291
C -4.469595883 -0.808591808 -3.453310677
H -7.699993378 -1.662108287 -2.700768443
C -5.594250087 -1.142435829 -2.657332437
C -3.223594360 -0.481057875 -2.790091576
N -4.151559863 -0.689162024 -0.547514915
C -3.710594368 -0.547196448 0.695542136
C -3.097554388 -0.421524104 -1.425634908
C -1.909153054 -0.072446490 -0.626929939
N -2.391380501 -0.177552553 0.711722035
B -5.568794118 -1.285368330 -1.047857734
F -5.585947884 -2.623768790 -0.607902811
F -6.578735061 -0.537997319 -0.407173139
C -1.568089285 0.086239384 1.884613296
H -1.939673715 0.965410960 2.434226054
H -1.547490767 -0.791784184 2.548382059
H -0.553654326 0.290315295 1.511147921
C -4.517993544 -0.786923446 1.921091424
H -5.579891523 -0.825454678 1.647309320
H -4.222473581 -1.746339326 2.380344620
H -4.346526539 0.016944633 2.654966702
O -0.764584052 0.244552220 -0.941295971
H -2.316229172 -0.244997787 -3.354933231
C -4.854055385 -0.699875862 -7.735679669
C -5.944815490 -1.018767170 -6.938470609
C -3.603065922 -0.402723982 -7.133256821
H -6.917080395 -1.249457750 -7.389192375

H -2.741041225 -0.151281735 -7.760097030
C -3.459517098 -0.430341409 -5.749486092
H -2.480415731 -0.193834806 -5.324028256
H -4.956298569 -0.674857416 -8.825679405

Compound **2B**
Energy = -1173.7022155113 Hartree
C -5.788409518 -1.053489121 -5.516525545
C -6.915336886 -1.378368357 -4.697971880
C -4.522758745 -0.730549213 -4.881456566
H -7.870925869 -1.604434409 -5.187234535
C -6.802338244 -1.420743311 -3.316470634
C -4.446067362 -0.798780404 -3.429373176
H -7.680698080 -1.671488664 -2.711040331
C -5.575223185 -1.138232679 -2.648915541
C -3.210917198 -0.463331256 -2.753359790
N -4.155922185 -0.695024225 -0.521184685
C -3.728075920 -0.559206439 0.727866923
C -3.096294567 -0.413872876 -1.386705736
C -1.918017331 -0.062887672 -0.575026776
N -2.411945057 -0.181682640 0.758859868
B -5.566492848 -1.288138464 -1.039616567
F -5.591137428 -2.629017940 -0.607724026
F -6.584076402 -0.543102051 -0.408177790
C -1.600908146 0.078432771 1.941084548
H -1.982089613 0.951998548 2.493069988
H -1.581977126 -0.803947823 2.599089929
H -0.584147415 0.290203768 1.578367508
C -4.546619856 -0.812033796 1.943343852
H -5.605602141 -0.852943528 1.658714524
H -4.251732534 -1.773659770 2.398389586
H -4.386265469 -0.013037042 2.685018691
O -0.772421416 0.263954508 -0.875942426
H -2.300358984 -0.217137316 -3.308928720
C -4.805469915 -0.685963859 -7.742282840
C -5.903688063 -1.009413416 -6.922010562
C -3.538087203 -0.368202280 -7.115704812
H -6.870169473 -1.249358724 -7.383734630
C -3.426788182 -0.412250437 -5.708687992
H -2.451779246 -0.165919477 -5.275695978
C -3.799655691 -0.312793470 -9.942977502
C -4.901742737 -0.641294728 -9.171534227
C -2.546616397 -0.006717981 -9.326340535
H -5.862772473 -0.876898525 -9.643575241
H -1.683678730 0.250532591 -9.949671943
C -2.419909030 -0.036766036 -7.947421030
H -1.459832089 0.198034144 -7.472305955
H -3.886557234 -0.284554755 -11.034490246

Compound **3B**
Energy = -1478.9942070639 Hartree
C -5.750021309 -1.061122179 -5.519576074
C -6.888114599 -1.381060171 -4.711943945
C -4.490761010 -0.731425985 -4.866032232
H -7.837930452 -1.608981543 -5.211593569
C -6.791354801 -1.420021271 -3.330340260

C -4.431717071 -0.799707032 -3.410091942
H -7.677539834 -1.667777230 -2.735128258
C -5.570985631 -1.135417981 -2.645957395
C -3.207118378 -0.462154472 -2.717597405
N -4.181791668 -0.692736479 -0.498528684
C -3.771422946 -0.556346494 0.756841271
C -3.110856097 -0.412302693 -1.349090064
C -1.943888666 -0.060906213 -0.521776758
N -2.456076228 -0.179265512 0.805580937
B -5.585317076 -1.283744699 -1.036594953
F -5.619236915 -2.624426587 -0.604661833
F -6.611123030 -0.536470586 -0.421191432
C -1.661067336 0.081217896 1.998598632
H -2.049405492 0.955249628 2.544819971
H -1.651478728 -0.800823282 2.657250719
H -0.639394578 0.292390016 1.649590279
C -4.607295115 -0.808160846 1.960659246
H -5.662230745 -0.847826827 1.661158905
H -4.320119697 -1.770121740 2.419945781
H -4.456546644 -0.009309167 2.704508102
O -0.794169990 0.265807889 -0.807068288
H -2.288875449 -0.216305052 -3.260645913
C -4.741058682 -0.704089614 -7.740349729
C -5.851667106 -1.020646574 -6.922518088
C -3.472867318 -0.374996143 -7.090359619
H -6.813104831 -1.264638933 -7.392906016
C -3.384529397 -0.421942173 -5.674637618
H -2.416130423 -0.173084985 -5.228046435
C -4.825365494 -0.662899634 -9.150325008
H -5.779940926 -0.902619761 -9.636836790
C -2.352899102 -0.054101488 -7.887829448
H -1.398465754 0.184844214 -7.399892290
C -2.660623161 0.028137490 -12.151481071
C -3.778478689 -0.300257384 -11.364424265
C -1.395139912 0.341871383 -11.504661997
H -4.731057161 -0.536710246 -11.856472579
C -3.706263075 -0.337598273 -9.948863858
C -1.312349534 0.310192038 -10.101639954
H -0.357349559 0.547349247 -9.614285351
C -2.432646854 -0.017612054 -9.298053486
C -1.615342181 0.391067013 -14.339028674
C -2.728090047 0.065037864 -13.585653570
C -0.368980854 0.699565860 -13.701863450
H -3.681054153 -0.170879921 -14.073983033
H 0.503204368 0.955834898 -14.312721810
C -0.263704359 0.675107342 -12.323382552
H 0.688811044 0.910817430 -11.833842742
H -1.684389764 0.414201999 -15.431959968

Compound **1C**
Energy = -1021.0537335528 Hartree

C -5.734187240 -1.194940606 -5.544242883
C -6.904911783 -1.503008543 -4.758826686
C -4.524753571 -0.895584796 -4.865699907
C -6.803913512 -1.510614338 -3.333337464

C -4.463714519 -0.910400692 -3.462880538
H -7.705203168 -1.748284967 -2.753461906
C -5.617835606 -1.235155766 -2.659509620
C -3.220183585 -0.541282608 -2.819475681
N -4.163946442 -0.683400800 -0.577420172
C -3.732321403 -0.487004844 0.659764426
C -3.103418493 -0.427678554 -1.459014404
C -1.927944293 -0.025605377 -0.663075753
N -2.418621856 -0.089578024 0.671766722
B -5.579738626 -1.339876771 -1.049359139
F -5.537889721 -2.661908036 -0.565120690
F -6.599035750 -0.606895288 -0.408947132
C -1.610513274 0.231998038 1.840694825
H -2.002568993 1.123672774 2.354831724
H -1.579888938 -0.620517320 2.536574203
H -0.596623381 0.440856837 1.468424132
C -4.536402790 -0.699980539 1.892544875
H -5.596481710 -0.774464144 1.620134553
H -4.217852788 -1.634314717 2.386854335
H -4.384637620 0.134298001 2.596223836
O -0.787782109 0.301331354 -0.983150737
H -2.330245660 -0.313608114 -3.420015499
H -3.628481634 -0.642959893 -5.447730471
C -7.034289895 -1.481366110 -7.594991513
C -8.190320952 -1.785361285 -6.822650491
C -5.828819188 -1.190624076 -6.968821886
H -4.935910897 -0.955111753 -7.559907539
C -8.125016747 -1.795586887 -5.433259751
H -9.014736236 -2.030299583 -4.837349843
H -9.135562082 -2.012999724 -7.327030502
H -7.097573234 -1.476395666 -8.688383905

Compound **2C**
Energy = -1173.7052487199 Hartree
C -5.730257851 -1.199611856 -5.548709973
C -6.913552558 -1.493328503 -4.758106118
C -4.516859041 -0.909617033 -4.863143221
C -6.807613105 -1.500409789 -3.326748664
C -4.459099042 -0.923677669 -3.463229296
H -7.711297198 -1.728131961 -2.746334088
C -5.621356964 -1.239194910 -2.656524681
C -3.217532196 -0.556545859 -2.819940448
N -4.166535097 -0.682539147 -0.576835902
C -3.736184149 -0.477013906 0.659475755
C -3.104533236 -0.434443440 -1.458845587
C -1.929978708 -0.026815761 -0.664715713
N -2.422311717 -0.079327649 0.669541763
B -5.577993777 -1.351244051 -1.047213166
F -5.518543147 -2.674902649 -0.568915120
F -6.603511921 -0.633124829 -0.400230625
C -1.615768307 0.252046475 1.836744257
H -2.008334606 1.148199911 2.342673062
H -1.586367630 -0.594433589 2.540023442
H -0.601291217 0.457460937 1.464144109
C -4.540125243 -0.682219261 1.893618975

H -5.599488678 -0.765703411 1.621146323
H -4.216384946 -1.609466394 2.397895403
H -4.393980445 0.160031813 2.589053319
O -0.789188369 0.297470703 -0.986577823
H -2.325403754 -0.334086405 -3.419231646
H -3.617230275 -0.664855475 -5.443687819
C -7.029423139 -1.474463118 -7.618453718
C -8.213230193 -1.769023769 -6.830678249
C -5.815470030 -1.194298741 -6.957992535
H -4.918668055 -0.969629588 -7.550257133
C -8.123244390 -1.772286595 -5.420837975
H -9.018906574 -1.996257755 -4.826933359
C -8.338956943 -1.752006410 -9.669990071
C -9.504080943 -2.043127188 -8.895086800
C -7.133049790 -1.474548489 -9.047923023
H -10.450996082 -2.260999080 -9.400495515
H -6.238615975 -1.251514903 -9.641536604
C -9.441034082 -2.051564085 -7.510999945
H -10.333966668 -2.274947186 -6.915253177
H -8.402620411 -1.748733739 -10.763426722

Compound **1D**
Energy = -1021.0529854980 Hartree
C -5.868605681 -1.109246699 -5.481774434
C -7.007415766 -1.485025320 -4.707260192
C -4.681584722 -0.769004904 -4.861238102
C -6.904969623 -1.506673866 -3.265059887
C -4.580688140 -0.792520706 -3.431649511
C -5.677257383 -1.156699018 -2.611244599
H -5.949735801 -1.093998855 -6.575118416
C -3.309010667 -0.423226559 -2.842726850
N -4.110038776 -0.704689659 -0.574429823
C -3.616746130 -0.588455794 0.651376977
C -3.102010515 -0.385806388 -1.489512642
C -1.885499496 -0.036672966 -0.734976320
N -2.303950063 -0.195960916 0.619619981
B -5.585757678 -1.224910677 -0.993216492
F -5.681447527 -2.531729067 -0.473434517
F -6.493457374 -0.367186386 -0.330802210
C -1.429655817 0.040486847 1.761106432
H -1.784204235 0.897140568 2.355647634
H -1.368237998 -0.857991887 2.394189719
H -0.436262830 0.269106678 1.347431136
C -4.364785858 -0.873819107 1.904973281
H -5.441129754 -0.847122235 1.693544342
H -4.094115848 -1.876095083 2.280212855
H -4.113185224 -0.130689836 2.677941433
O -0.762256030 0.311771535 -1.089647879
H -2.461818821 -0.155440957 -3.486646631
H -3.805955453 -0.479784281 -5.454182960
C -8.236932582 -1.840653547 -5.332161806
C -9.339972354 -2.207522438 -4.571147855
C -9.246901113 -2.229073514 -3.152391208
C -8.062729760 -1.886958174 -2.512259127
H -8.004755251 -1.900027806 -1.421603122

H -8.298482882 -1.819807892 -6.426796985
H -10.119956077 -2.517129614 -2.557051328
H -10.280436514 -2.479431373 -5.062544426

Compound **2D**
Energy = -1173.7054760559 Hartree
C -5.862772490 -1.115126724 -5.487434083
C -7.012161260 -1.485961988 -4.714794078
C -4.681024050 -0.776187392 -4.865945297
C -6.909510519 -1.504618904 -3.262797814
C -4.580485508 -0.796846681 -3.429401027
C -5.675026590 -1.154527059 -2.609810436
H -5.940838290 -1.102680806 -6.581167382
C -3.309723524 -0.428317880 -2.841431669
N -4.109268687 -0.704322926 -0.572283891
C -3.616516345 -0.584216020 0.654339304
C -3.102012547 -0.387445959 -1.487405291
C -1.886253723 -0.034904994 -0.734409822
N -2.304940516 -0.190166647 0.621287459
B -5.581137449 -1.231644417 -0.991788357
F -5.668743001 -2.542933814 -0.482387474
F -6.495252341 -0.385258762 -0.322626499
C -1.430780941 0.051091055 1.761865243
H -1.787312052 0.908247558 2.354485951
H -1.366930548 -0.845727026 2.397073450
H -0.438152663 0.281178914 1.347160547
C -4.364178993 -0.868767027 1.908322366
H -5.440504099 -0.846432028 1.696287665
H -4.090073124 -1.869034588 2.286567679
H -4.115686605 -0.122717759 2.679524449
O -0.763039956 0.313874283 -1.089428617
H -2.462203889 -0.162309175 -3.485644987
H -3.802936016 -0.491088552 -5.457161555
C -8.225022890 -1.836385808 -5.336501614
C -9.360757113 -2.204034478 -4.585147220
C -9.266578261 -2.220178200 -3.137942844
C -8.053691147 -1.868442307 -2.512167856
H -7.999839119 -1.873657542 -1.419765063
H -8.288658890 -1.819701700 -6.432600594
C -10.601024555 -2.563459838 -5.203671356
C -11.698102757 -2.919198693 -4.436307233
H -12.641053983 -3.191666117 -4.922461922
C -11.606718581 -2.933465091 -3.009377852
H -12.481876755 -3.216536692 -2.414576531
C -10.423421132 -2.592466336 -2.376441900
H -10.350493979 -2.603089798 -1.282763372
H -10.668429390 -2.551362343 -6.298141045
